# Structure‐activity relationship of the peptide binding‐motif mediating the BRCA2:RAD51 protein–protein interaction

**DOI:** 10.1002/1873-3468.12139

**Published:** 2016-04-06

**Authors:** Duncan E. Scott, May Marsh, Tom L. Blundell, Chris Abell, Marko Hyvönen

**Affiliations:** ^1^ Department of ChemistryUniversity of CambridgeCambridgeUK; ^2^ Department of BiochemistryUniversity of CambridgeCambridgeUK; ^3^ Present address: Paul Scherrer InstituteVilligenSwitzerland

**Keywords:** alanine scanning, biophysics/ITC, peptides, protein–protein interaction, RAD51, X‐ray crystallography

## Abstract

RAD51 is a recombinase involved in the homologous recombination of double‐strand breaks in DNA. RAD51 forms oligomers by binding to another molecule of RAD51 via an ‘FxxA’ motif, and the same recognition sequence is similarly utilised to bind BRCA2. We have tabulated the effects of mutation of this sequence, across a variety of experimental methods and from relevant mutations observed in the clinic. We use mutants of a tetrapeptide sequence to probe the binding interaction, using both isothermal titration calorimetry and X‐ray crystallography. Where possible, comparison between our tetrapeptide mutational study and the previously reported mutations is made, discrepancies are discussed and the importance of secondary structure in interpreting alanine scanning and mutational data of this nature is considered.

## Abbreviations

**BRCA2**, breast cancer type‐2 susceptibility protein

**HR**, homologous recombination

**ITC**, isothermal titration calorimetry

**PPI**, protein–protein interaction

**SAR**, structure activity relationship

Eukaryotic RAD51, archeal RadA and prokaryotic RecA are a family of ATP‐dependent recombinases involved in homologous recombination (HR) of double‐strand breaks in DNA 1. RAD51 interacts with BRCA2, and is thought to localise RAD51 to sites of DNA damage 2, 3. Both BRCA2 and RAD51 together are vital for helping to repair and maintain a high fidelity in DNA replication. BRCA2 especially has garnered much attention in a clinical context, as many mutations have been identified that drive an increased risk of cancer in individuals 4, 5. Although the inactivation of the BRCA2:RAD51 DNA repair pathway can cause genomic instability and eventual tumour development, an inability to repair breaks in DNA may also engender a sensitivity to ionising radiation 6, 7. For this reason it is hypothesised that in tumour cells with an intact BRCA2:RAD51 repair pathway, small molecules which prevent the interaction between RAD51 and BRCA2 may confer radiosensitivity by disabling the HR pathway 8. The interaction between the two proteins is mediated by eight BRC repeats, which are characterised by a conserved ‘FxxA’ motif 9. RAD51 and RadA proteins also contain an ‘FxxA’ sequence (FTTA for human RAD51) through which it can bind to other RAD51 and RadA molecules, and oligomerise to form higher order filament structures on DNA. The common FxxA motifs of both the BRC repeats and RAD51 oligomerisation sequence are recognised by the same FxxA‐binding site of RAD51.

In general, the dominant contribution of certain residues to the overall binding energy of a protein–protein interaction are known as ‘hot‐spot’ residues. Interestingly, small molecule inhibitors of PPIs are often found to occupy the same pockets which are otherwise occupied by hot‐spot residues in the native complex. It is therefore of great interest to identify hot‐spots in an effort to guide drug discovery efforts against a PPI. Further, a correlation between residues that are strongly conserved and hot‐spot residues has been reported 10. Purely based on the amino acid consensus sequence reported by Pellegrini *et al*., 11 phenylalanine and alanine would both be expected to be hot‐spots and to a lesser extent, threonine. However, whilst the identification of highly conserved residues may be a good starting point for identifying hot‐spots, experimental validation by mutation of these sequences is vital.

The importance of residues in the FxxA motif has been probed by a variety of techniques, collated in Table 1. Briefly, mutating phenylalanine to glutamic acid inactivated the BRC4 peptide and prevented RAD51 oligomerisation 11, 12. A phenylalanine‐truncated BRC4 is also found to be inactive 13, however, introducing a tryptophan for phenylalanine was found to have no significant effect on BRC4 affinity 12. A glutamine replacing the histidine in BRC4 maintains BRC4 activity 13. The ability of BRC3 to interact with RAD51 nucleoprotein filaments is disrupted when threonine is mutated to an alanine 3. Similarly, mutating alanine to glutamic acid in the RAD51 oligomerisation sequence 11 or to serine in BRC4 13 leads to loss of interaction in both cases. The BRC5 repeat in humans has serine in the place of alanine, and is thought to be a nonbinding repeat 12. Mutations identified in the clinic, in the FxxA region of the BRC repeats of BRCA2 are collated in Table 1 14. It is difficult to state the clinical relevance of these mutations as they are annotated as ‘unvalidated’, that is, it is not known whether they contribute to the disease phenotype or are neutral variants. For completeness, we present them here with this caveat, and to make the comment that these clinical mutations are consistent with abrogating the interaction with RAD51.

**Table 1:** Summary of FxxA‐relevant mutations previously reported and degree of characterisation. The mutation, relevant peptide context, resulting FxxA motif sequence and experimental technique for each entry is given. For clarity, mutated residues are shown in bold

Mutation context^a^	Mutation	FxxA motif	Technique used	Effect
RAD51 (FTTA)	F86E	**E**TTA	Immunoprecipitation 11	No binding
BRC4 (FHTA)	F1524E	**E**HTA	Competitive ELISA 12	Peptide inactive
BRC4 (FHTA)	F1524W	**W**HTA	Competitive ELISA 12	Comparable activity to WT
BRC4 (FHTA)	F1524V	**V**HTA	BRCA2 mutations database 14	–
BRC4 (FHTA)	ΔF1524	**‐**HTA	Dissociation of RAD51‐DNA complex 13	Peptide inactive
BRC4 (FHTA)	H1525Q	F**Q**TA	Dissociation of RAD51‐DNA complex 13	Comparable activity
BRC7 (FSTA)	S1979R	F**R**TA	BRCA2 mutations database 14	–
BRC3 (FQTA)	T1430A	FQ**A**A	RAD51:DNA bandshift assay 3	Peptide inactive
BRC3 (FQTA)	T1430A	FQ**A**A	Electron microscopic visualisation of nucleoprotein filaments 3	Peptide inactive
BRC1 (FRTA)	T1011R	FR**R**A	BRCA2 mutations database 14	–
BRC2 (FYSA)	S1221P	FY**P**A	BRCA2 mutations database 14	–
BRC2 (FYSA)	S1221Y	FY**Y**A	BRCA2 mutations database 14	–
RAD51 (FTTA)	A89E	FTT**E**	Immunoprecipitation 11	No binding
BRC4 (FHTA)	A1527S	FHT**S**	Dissociation of RAD51‐DNA complex 13	Peptide inactive

a The wild‐type FxxA sequence is indicated in parenthesis.

In this work, we report the most detailed study of systematic mutations of peptides to probe the FxxA‐binding motif to date. We have chosen to focus on tetrapeptides, which allows us to examine the effect of mutation on the fundamental unit of binding, FxxA, rather than in the context of either the BRC repeat or self‐oligomerisation sequence. Affinities of peptides were measured directly using Isothermal Titration Calorimetry (ITC) and the structures of many of the peptides bound to humanised RadA were determined by X‐ray crystallography. The use of ITC is generally perceived as a gold‐standard in protein–ligand characterisation, rather than a competitive assay which may be prone to aggregation artefacts. Wild‐type human RAD51, however, is a heterogeneous mixture of oligomers and when monomerised by mutation, is highly unstable. In this context, we have previously reported the use of stable monomeric forms of RAD51, humanised from *Pyrococcus furiosus* homologue RadA, for ITC experiments and X‐ray crystallography 8, 15.

## Materials and methods

### Peptide synthesis

Peptides were synthesised using solid‐phase FMOC chemistry by Alta Biosciences (Birmingham, UK) or the Protein and Nucleic Acid Service at the Department of Biochemistry (University of Cambridge). All peptides prepared and used in the study were N‐acetylated and C‐amide terminated.

### Protein preparation

Protein expression and purification was performed as described previously 15. In brief, monomeric HumRadA2 was expressed in *E. coli* using T7‐based expression vector at 37 °C for 3 h. Soluble cell lysate was heat treated to precipitate most of the cellular proteins and the soluble fraction containing HumRadA2 was purified using a combination of cation exchange chromatography at pH 6.0 and size‐exclusion chromatography in 10 mm MES, 100 mm NaCl pH 6.0 buffer. Protein concentration was determined using the calculated extinction coefficient at 280 nm, and stored at −80 °C in small aliquots after flash freezing.

### Isothermal titration calorimetry

Isothermal titration calorimetry experiments were performed at 25 °C on a MicroCal iTC200. HumRadA2 (600 μm in 20 mm MES pH 6.0 with 100 mm NaCl and 0.5 mm EDTA) was diluted with Tris buffer (200 mm, pH 7.5 with 100 mm NaCl) to 64–83 μm. Peptides were dissolved in MilliQ water (50 mm) and an aliquot taken and diluted with 200 mm Tris, pH 7.5, 100 mm NaCl to give a ligand solution of 2.5–5 mm. The peptide solution was titrated into the protein solution; 16 injections (2.4 μL) of 4.8 s duration were made at 80‐s intervals. The initial injection of ligand (0.4 μL) was discarded during data analysis. Control experiments of peptides to buffer showed insignificant heats. The data were processed and thermodynamic parameters obtained by fitting the data to a single‐site‐binding model using Origin software and fixing the stoichiometry as 1.0 for weak‐binding ligands 16. All data from ITC measurements are shown in the Figs S1 and S2.

### X‐ray crystallography

Monomerised RadA proteins were crystallised in the same conditions as described previously 15. Peptides were soaked into the crystals at 2–5 mm concentration overnight in the presence of 10% glycerol as a cryoprotectant. Crystals were cryo‐cooled in liquid nitrogen and data collected at synchrotron light sources and processed using XDS: details of this are found in crystallographic table (Table S1 in Supporting Information). Structures were solved by molecular replacement using unliganded, monomeric RadA coordinates (PDB: 4b3b, after removal of FHTA peptide) as a search model and refined with an automated procedure using Refmac5 17. After inspection of the resulting electron density, the bound peptides were modelled into the density and structures were further refined using Refmac5 18 and phenix.refine 19, and manually rebuilt using Coot 20. Coordinates and structure factors have been deposited in the PDB under accession codes as listed in Table 2 and in the crystallographic data table in the Supporting Information. With the exception of FATA peptide complex, which was crystallised with wild‐type RadA, the structures are determined using HumRadA1 mutant.

**Table 2:** Summary of peptide‐binding data determined by ITC against HumRadA2. Mutated residues are highlighted in bold. All peptides were N‐acetylated and C‐amide terminated

Table entry	Peptide	*K*_D_/μm	Δ*H*/cal·mol^−1^	*T*Δ*S*/cal·mol^−1^	PDB code
First position variation					
1	FHTA	280 ± 20	−2388 ± 94	2453	4b3b 15
2	**W**HTA	93 ± 3	−2768 ± 34	2727	5fow
Second position variation					
3	F**A**TA	280 ± 29	−1820 ± 109	3010	5fpk a
4	F**N**TA	613 ± 44	−4036 ± 177	346	–
5	F**P**TA	No detectable binding			–
Third position variation					
6	FH**P**A	113 ± 3	−2155 ± 26	3218	5fou
7	FH**A**A	675 ± 60	−7948 ± 466	−3636	5fox
Fourth position variation					
8	FHT**G**	1590 ± 300	−5518 ± 924	−1702	5fov
9	FHT**U**	680 ± 51	−14 600 ± 771	−10 281	5fot
Combination					
10	**W**H**P**A	330 ± 25	−6801 ± 318	−2044	–
Peptide truncations					
11	FH	No binding detected No binding detected No binding detected			–
12	FHT				–
13	HTA				–

a Structure solved with wild‐type RadA.

### Sequence analysis

**Figure 1:**
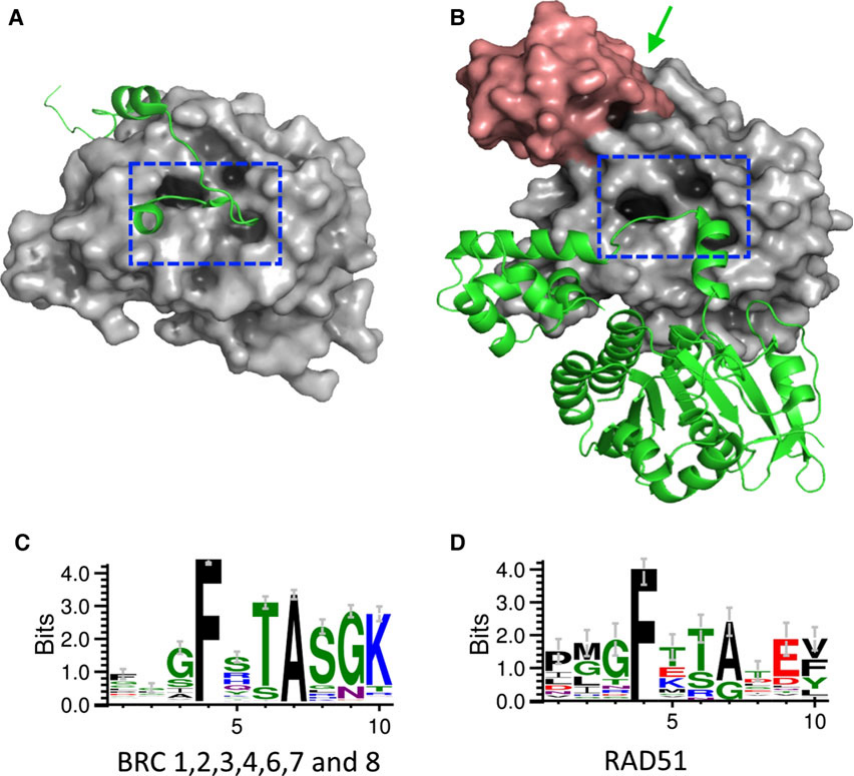
Conservation of FxxA motif (A) BRC4 peptide (green cartoon) bound to truncated human RAD51 (grey surface) (PDB: 1n0w, 11). The blue dashed box highlights the FxxA interaction pocket. (B) Two interacting protein molecules of RAD51 from *Saccharomyces cerevisiae* are shown. One RAD51 (green cartoon) interacts with another molecule of RAD51 (grey and pink surface) via the FxxA pocket indicated by the dashed blue box. The N‐terminal domain of one RAD51 protomer is highlighted in pink for clarity and the green arrow indicates the location of this protomer's FxxA oligomerisation sequence (PDB: 1szp, 29). (C) Conservation of FxxA motif across the human BRC repeats and (D) across 21 eukaryotic RAD51s and 24 RadAs, with the size of the letters proportional to the degree of conservation. Sequence figures generated using Weblogo 3.0 21, sequence details are found in the Supporting Information.

Sequences of mammalian RAD51 proteins and archeal RadA orthologues were obtained from Ensembl (www.ensembl.org) and Uniprot (www.uniprot.org) databases. Sequences were aligned using ClustalX2, and aligned sequences for the FxxA motifs were used in WebLogo (weblogo.berkely.edu/logo.cgi) server 21 to derive the consensus diagrams shown in Figs 1 and 4. All the sequences used in these analyses are shown in Figs S4, S5 and S6.

## Results

We have mutated and truncated the tetrapeptide epitope FHTA, and examined the effects both structurally and on the binding affinity with humanised RadA. As a comparative reference, we are using the FHTA sequence derived from the most tightly binding BRC repeat, BRC4 22. The peptides used are N‐acetylated and C‐amide terminated in order to provide the most relevant peptide in the context of a longer peptide chain. A summary of the peptide sequence, PDB codes and *K*_D_ data measured by ITC with the corresponding Δ*H* and *T*Δ*S* values are collated in Table 2.

Phe1524 of BRC4 binds in a small surface pocket of human RAD51, defined by the hydrophobic side chains of residues Met158, Ile160, Ala192, Leu203 and Met210. The residue is highly conserved across BRC repeats and oligomerisation sequences. Consistent with this, the truncated HTA tripeptide could not be detected to bind to humanised, monomeric RadA, HumRadA2 (Table 2, entry 13). As previously discussed, there is some evidence that substituting a tryptophan for the phenylalanine at this position was tolerated in the context of BRC4 12. Therefore, the WHTA peptide was tested and found to not only be tolerated, but to increase the binding affinity of the peptide approximately threefold.

The second position of the tetrapeptide was found to be largely invariant to changes in the side chains that were investigated. The residue makes no interactions with the RAD51 protein, but may make an internal hydrogen bond with Thr1520 in the context of BRC4, Fig. 3A. Replacing the histidine with an asparagine, chosen to potentially mimic the hydrogen bond donor–acceptor nature of histidine, resulted in a moderate, twofold decrease in potency (Table 2, entry 4). Mutating to an alanine, recapitulated the potency of FHTA, implying that the interactions made by histidine do not contribute overall to binding affinity (Table 2, entry 3). FPTA was also tested, but was found to have no affinity for the protein (Table 2, entry 5). Modelling suggests that a proline in the second position would be expected to clash sterically with the surface of the protein, and provides a rationale for the lack of binding observed.

Threonine was mutated to an alanine, resulting in only a moderately weaker *K*_D_ (twofold, Table 2, entry 7). In the context of a tetrapeptide at least, this result implies a lack of importance of a threonine at this position. Interestingly, it was found that a proline at this position improved the affinity almost threefold, to 113 μm (Table 2, entry 6). This beneficial mutation was incorporated with another previously identified variant to produce the peptide WHPA. Disappointingly, the combined effect of the mutations was not additive and the potency was weakened to 690 μm.

While the importance of the phenylalanine may be possible to predict from examination of the crystal structure, the alanine appears to be of much less importance in this regard. It is, however, a highly conserved residue and clearly of interest for systematic mutation. Removing the alanine residue entirely produced the truncated tripeptide FHT, which did not bind (Table 2, entry 12). The unnatural amino acid, α‐amino butyric acid (U), was introduced at the fourth position, positioning an ethyl group into the alanine pocket (Table 2, entry 9). Perhaps surprisingly, it was accommodated and the affinity dropped only by twofold as compared to FHTA. The effect of simply removing the β‐carbon of alanine, by mutation to glycine (FHTG), produced an approximately sixfold drop in binding affinity (Table 2, entry 8). This is in line with the observation that alanine is not 100% conserved and some archeal RadA proteins contain a glycine in the place of alanine 23.

### Structural characterisation of peptide complexes

Structures of the key tetrapeptides were solved by soaking into crystals of a humanised form of RAD51, HumRadA1, which we have previously reported as a convenient surrogate system for RAD51 crystallography 15. The corresponding PDB codes are indicated in Table 2 and crystallographic data are found in the Supporting Information. All structures are of high resolution (1.2–1.7 Å) and the electron density for the peptide was clearly visible after the first refinement using unliganded RadA coordinates (Fig. S1).

**Figure 2:**
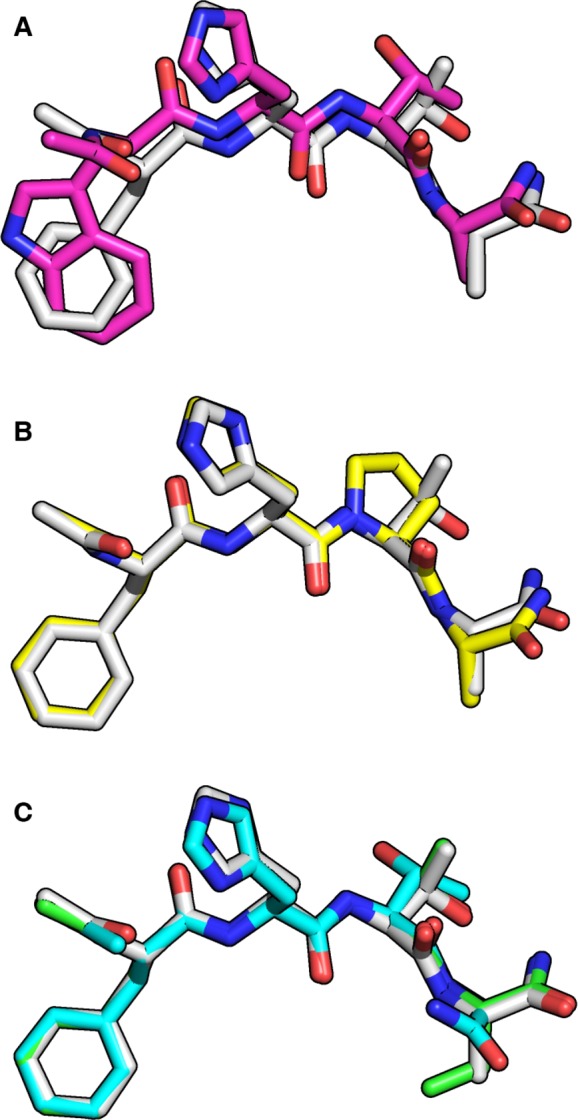
Comparison of different peptide complexes (A) Overlay with FHTA (grey) and WHTA (purple) showing a small relative displacement of the peptide backbone. (B) Superposition of FHTA (grey) and FHPA (yellow), showing conservation of backbone orientation (C) Overlay of FHTU (green), FHTA (grey) and FHTG (cyan).

Some of the SAR observed in the binding analysis can be interpreted in terms of these X‐ray crystal structures. For example, an overlay of the bound poses of the ligands FHTA and FHPA (Fig. 2B) reveals a high similarity in the binding modes, indicating that the conformational rigidity conferred by the proline is compatible with the FHTA‐binding mode, and a reduction in an entropic penalty of binding may be the source of the improvement in affinity. WHTA peptide shows a relative dislocation when compared to FHTA (Fig 2A), with the entire ligand backbone of WHTA shifted to accommodate the change in the position of the main chain carbon of the first residue, as the larger indole side chain fills the Phe pocket. This shift is translated all the way to the alanine side chain. It is possible that this mutation is beneficial in the tetrapeptide context and neutral in the full‐length BRC4 context because the smaller peptide is less constrained and allowed to explore more conformations. An attempt to combine both the tryptophan and proline mutations, however, led to no improvement for WHPA peptide compared to FHTA. One possible explanation is that the ‘shifted’ binding mode observed in WHTA was not compatible with the conformational restriction that the proline of WHPA introduced.

The thermodynamic data of peptide binding are also shown in Table 2. Although we have both thermodynamic data and high‐quality X‐ray structural information for some of the mutant peptides, we do not attempt to interpret differences in thermodynamic profiles between ligands, that is, to analyse ΔΔ*H* and ΔΔ*S*. Although Δ*H* and Δ*S* are tabulated, the *K*_D_s measured are relatively weak and necessarily performed under low c‐value conditions. In this experimental regime, nonsigmoidal curves are generated and therefore errors in Δ*H* are expected to be much higher than the errors from model fitting given in Table 2 16. As Δ*S* is derived from ΔG by subtracting Δ*H*, errors in Δ*H* will be correlated with errors in Δ*S*, giving rise to a ‘phantom’ enthalpy–entropy compensation. Such effects have been discussed by Klebe 24 and Chodera and Mobley 25 and will frustrate attempts to interpret the measured ΔΔ*H* and ΔΔ*S*.

### Understanding mutations, residue conservation and epitope secondary structure

The conserved phenylalanine and alanine residues of the FHTA sequence were both found to be essential for binding by ITC. Conversely the second position histidine residue, corresponding to the unconserved His1525 in the BRC4 sequence, could be mutated without significant effect on the peptide affinity. The more general correlation between hot‐spot residues in protein–protein interactions and the high conservation of such residues has been previously reported 10, 26. Interestingly, however, the highly conserved threonine residue could be mutated without affecting the peptide affinity. This unexpected result, in the light of its very high conservation in the BRC and oligomerisation sequences, begs the question of what the role of Thr1526 is and highlights a potential pitfall and need for caution in the experimental design of alanine mutation studies.

**Figure 3:**
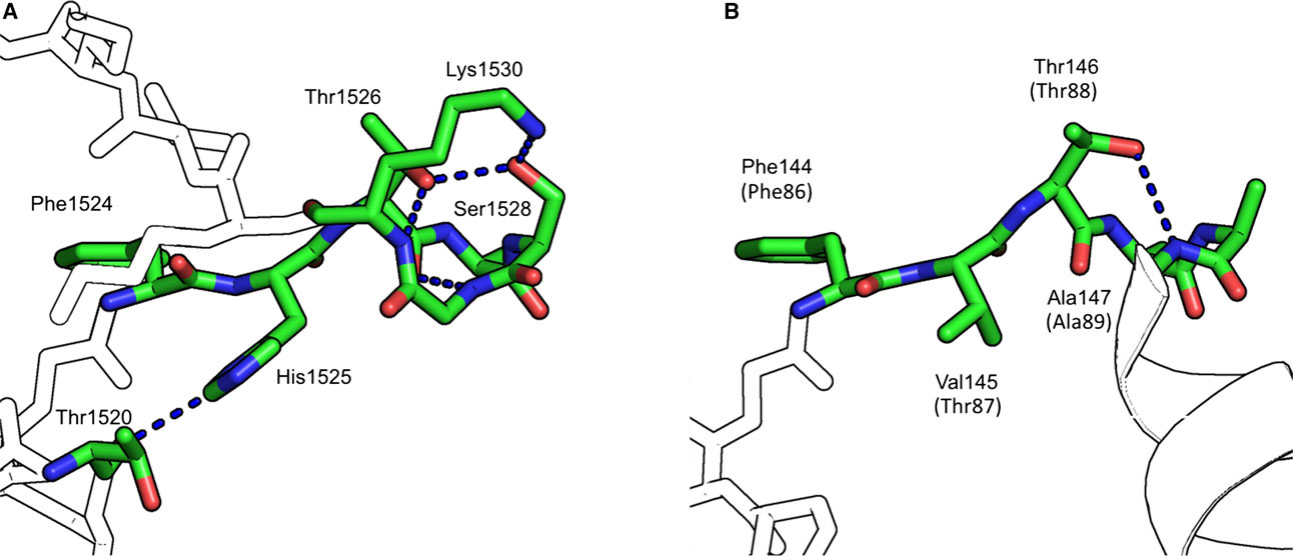
(A) Highlight of intra‐BRC4 interactions when bound to RAD51 (omitted for clarity) (PDB: 1n0w), with key residues shown in colour. (B) Intrapeptide interactions from oligomerisation epitope of *S. cerevisiae* RAD51 when bound to next RAD51 in the filament (PDB: 1szp). Colouring as in (A). Residue numbering relates to the *S. cerevisiae* RAD51 protein, the corresponding human residues are in parentheses.

As the FHTA peptide is potentially a surrogate peptide for both the BRC repeat peptides and the RAD51 self‐oligomerisation peptide, it is useful to examine the role of Thr1526 (BRC4) and the analogous Thr87 (RAD51) in both binding contexts in more detail. Structural information for these two interactions is limited. Only one structure of BRC4 is published in complex with human RAD51 (PDB: 1n0w). Figure 3A shows the binding pose of BRC4 when bound to RAD51 and the intrapeptide hydrogen bonds that are made by BRC4. While Phe1524 and Ala1527 are buried in hydrophobic pockets on the surface, His1525 is close enough to form a hydrogen bond with the carbonyl of Thr1520, but the rotamer of His1525, supported by clearly positioned water molecules, is not compatible with hydrogen bonding. Also, Thr1520 is constrained by crystal contacts in this structure. Lack of conservation of this residue supports the idea that this interaction is not crucial for RAD51:BRC repeat binding.

Either a threonine or serine is most commonly found in the third position of the FxxA motif. Thr1526 makes no direct interactions with the RAD51 protein, but instead forms a hydrogen bond network with the highly conserved S1528 and K1530 (Fig. 1C). The high degree of conservation of these three residues suggests an important possible role in facilitating a turn and stabilising the conformation of the peptide as it continues its way to a second interaction site on the side of RAD51. With respect to understanding the RAD51:RAD51 interaction, no human crystal structure has been published, however, several oligomeric structures of archaeal RadA as well that of *Saccharomyces cerevisiae* RAD51 have been reported 27, 28, 29. Figure 3B shows a highlight of the FxxA portion of oligomerisation peptide from the *S. cerevisiae* RAD51 structure, with residues in parentheses corresponding to the human RAD51 protein. The conserved threonine residue at the third position forms a hydrogen bond with the peptide backbone amide, which forms the base of an α‐helix.

In both structural contexts, the role of the third position threonine in FxxA seems to be in stabilising secondary structure; a β‐turn in the case of BRC binding and an α‐helix in the case of RAD51 oligomerisation. In the tetrapeptide context these secondary interactions are not present and mutation of threonine to alanine would be expected to have little effect on affinity. In line with this, although we observe a slight twofold weakening of peptide affinity, the effect is far from being as drastic or inactivating as reported in longer peptide backgrounds 3. It would be interesting to investigate the importance of this residue in the context of the BRC4 peptide, and the oligomerisation peptide. Rather than indifference to alanine mutation, a significant effect, via lack of secondary structure stabilisation, would be predicted, as indeed has been reported for BRC3 3.

## Conclusions

**Figure 4:**
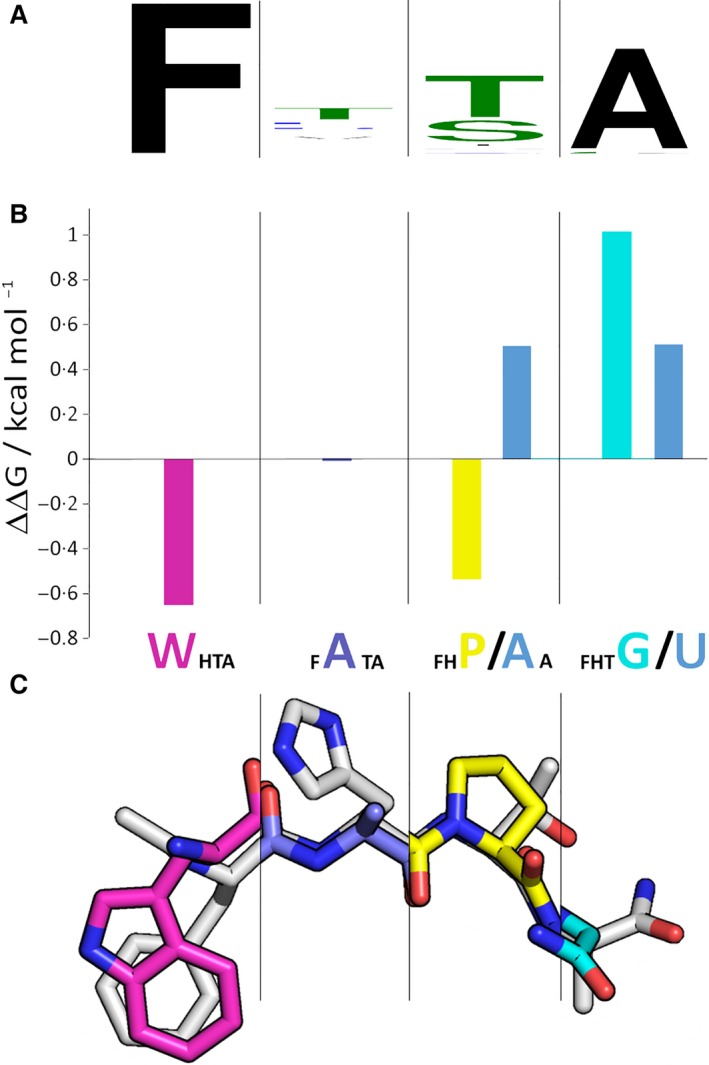
Summary of key observations (A) FxxA motif sequence conservation of Rad51 oligomerisation sequences and BRC repeats. (B) Highlight of SAR identified for the tetrapeptide. The differences in ΔG for different peptide variants relative to FHTA are shown in the bar chart with colouring matching with the structural overlay below. (C) Overlay of tetrapeptide structures, with wild‐type FHTA peptide across the figure for reference and truncated segments of mutated residues shown in each panel. Purple carbon is WHTA, light blue is FATA, yellow is FHPA, cyan is FHTG and grey carbon is FHTA. Note the C‐terminal amide changes position in FHTG without the anchoring methyl group.

The key observations from this work are shown in Fig 4. Two residues in the FxxA motif, phenylalanine and alanine, are highly conserved (Fig 4a). Phenylalanine mutated to tryptophan, in the context of the tetrapeptide improved potency, contrary to the reported result of comparable activity in the context of BRC4 12. Proline at the third position similarly improved potency. Activity was lost by mutating the terminal alanine to glycine, but recovered somewhat with the novel α‐amino butyric acid (U). Threonine was found to be relatively unimportant in the tetrapeptides but has been previously reported to be crucial in the context of BRC3. The reason for this disconnection is suggested to be that threonine plays a role in stabilising the β‐turn in the BRC repeats, which is absent in the tetrapeptides studied. This may lead to a more general caution, that hot‐spot data should be interpreted by considering the bound interaction with the protein, as well as the potential role in stabilising the bound peptide secondary structure. In either case, the requirement for structural data in correctly interpreting alanine‐scanning experiments is reinforced.

## Supporting information


**Fig. S1.** ITC data for Table 2 entries 1–9.
**Fig. S2.** ITC data for Table 2 entries 10–13.
**Fig. S3.** Sequence alignments used for consensus diagram in Fig. 1C for BRC repeats 1,2,3,4,6,7 and 8, along with consensus diagrams for each individual BRC repeat.
**Fig. S4.** BRC5 sequences and consensus alignment.
**Fig. S5.** Sequences used in Fig. 1D consensus diagram of RAD51/RadA oligomerisation motif.
**Fig. S6.** Sequences used in Fig. 4A consensus diagram.
**Table S1.** Crystallographic data collection, refinement and structure analysis.
