# Supplementary material for: Structure‐activity relationship of the peptide binding‐motif mediating the BRCA2:RAD51 protein–protein interaction
Source: FEBS Lett. 2016 Apr 6;590(8):1094–102. doi: 10.1002/1873-3468.12139 (PMC4855620; doi:10.1002/1873-3468.12139)
Supplement: Supplementary file 1 — Fig. S1. ITC data for Table 2 entries 1–9. Fig. S2. ITC data for Table 2 entries 10–13. Fig. S3. Sequence alignments used for consensus diagram in Fig. 1C for BRC repeats 1,2,3,4,6,7 and 8, along with consensus diagrams for each individual BRC repeat. Fig. S4. BRC5 sequences and consensus alignment. Fig. S5. Sequences used in Fig. 1D consensus diagram of RAD51/RadA oligomerisation motif. Fig. S6. Sequences used in Fig. 4A consensus diagram. Table S1. Crystallographic data collection, refinement and structure analysis. [file FEB2-590-1094-s001.docx]

## Supplementary material for Scott *et al.* “Structure Activity Relationship of the Peptide Binding Motif Mediating RAD51:BRCA2 Protein-Protein Interaction”

### Supplementary figure 1: ITC data for Table 2 entries 1-9


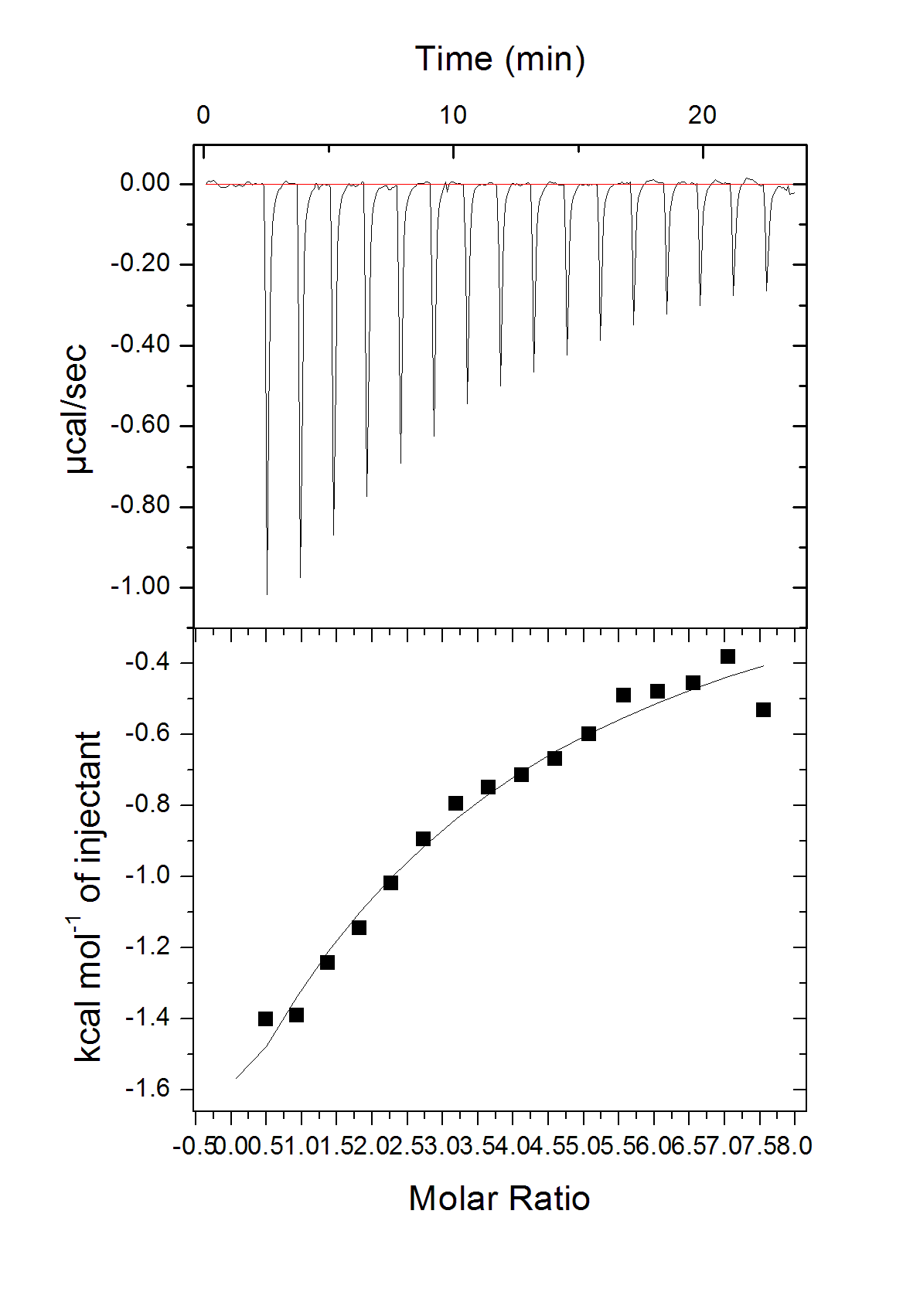

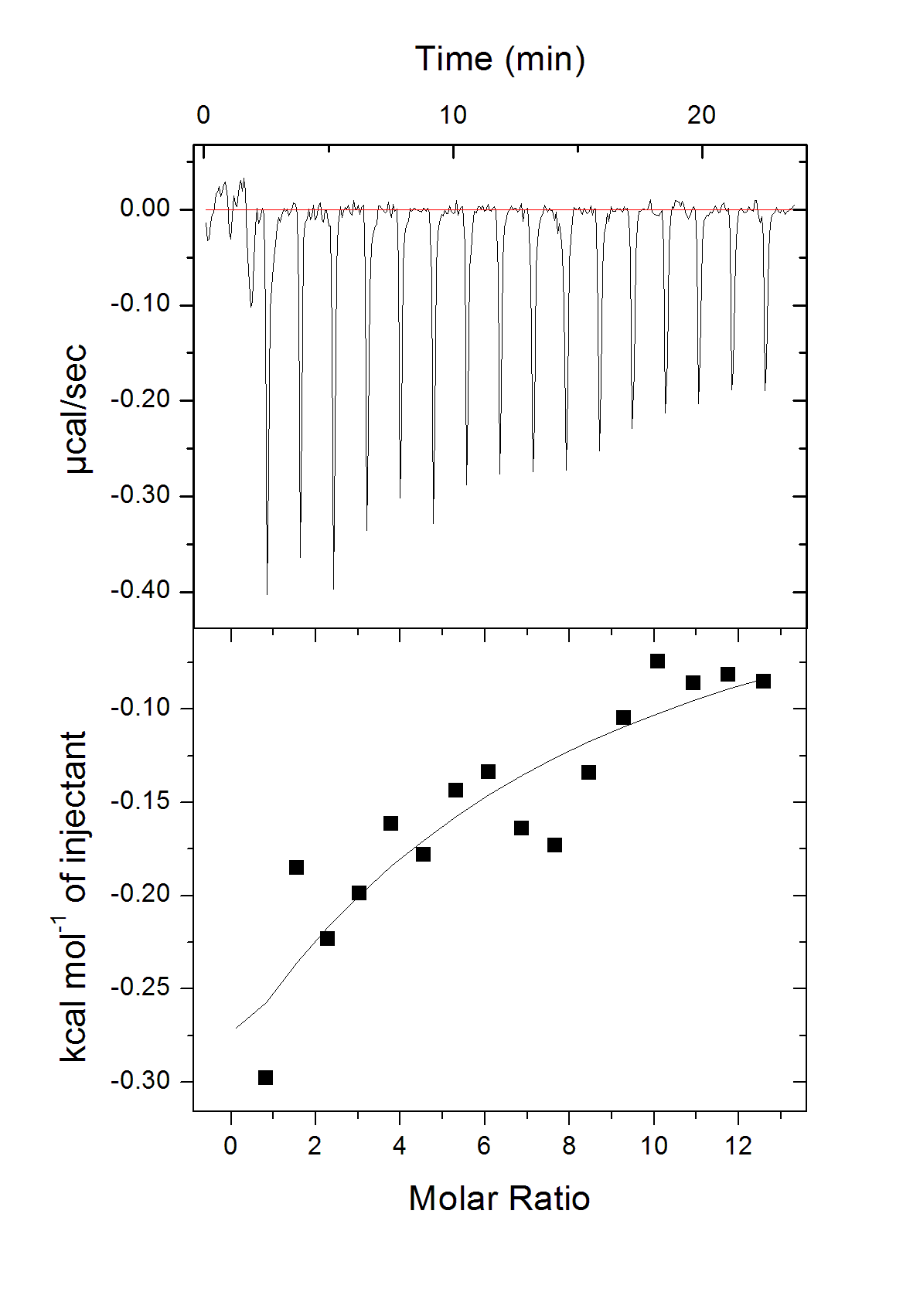

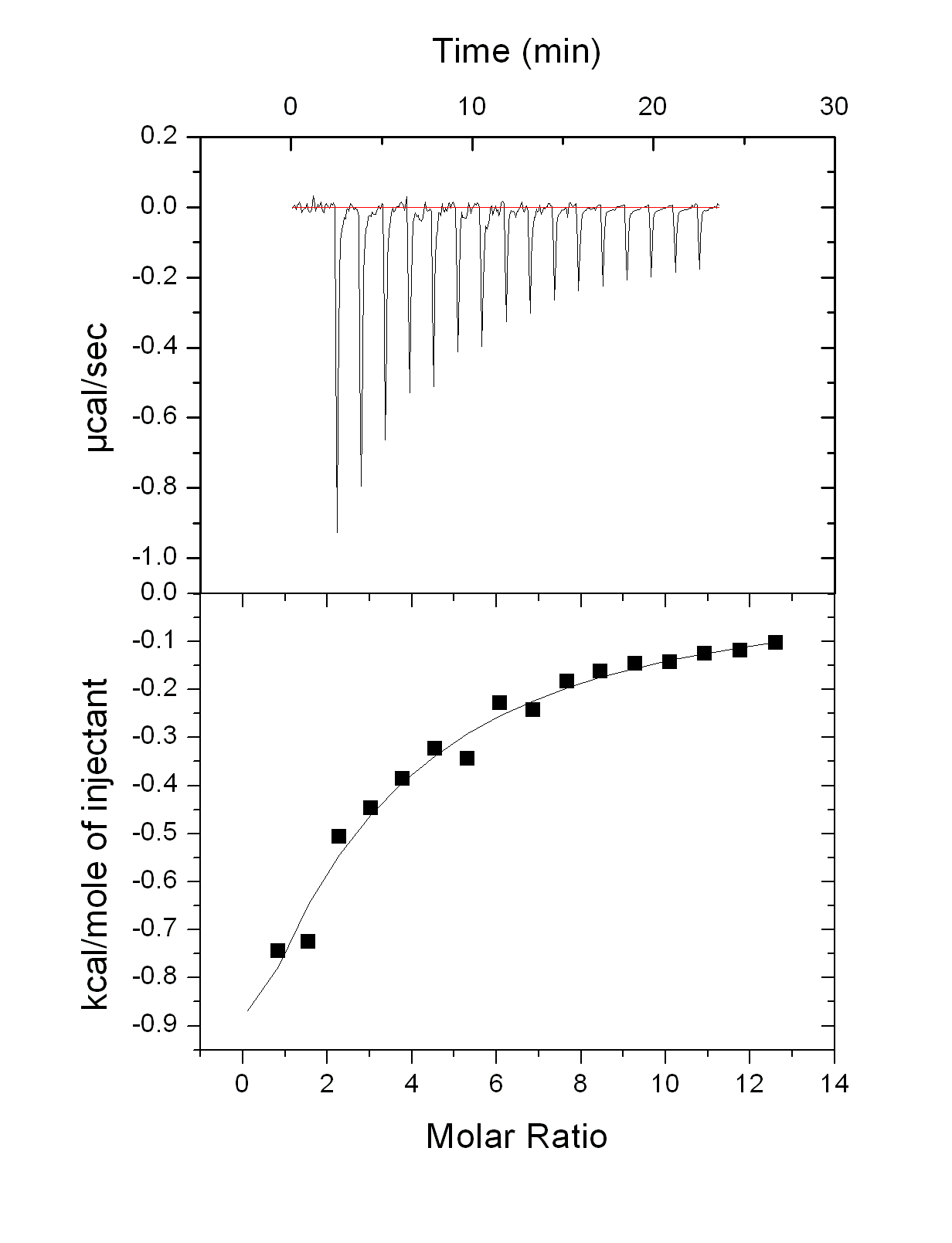

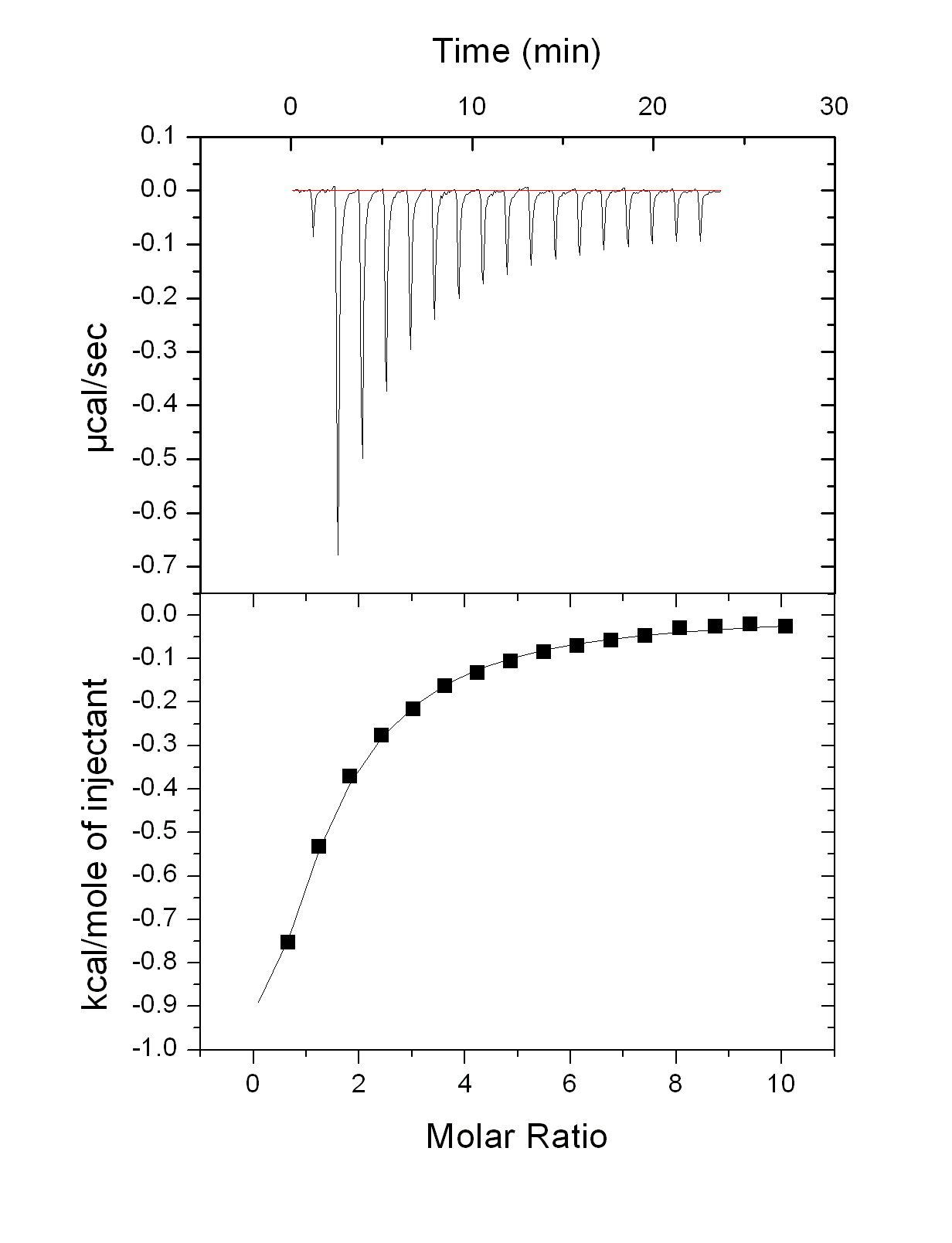

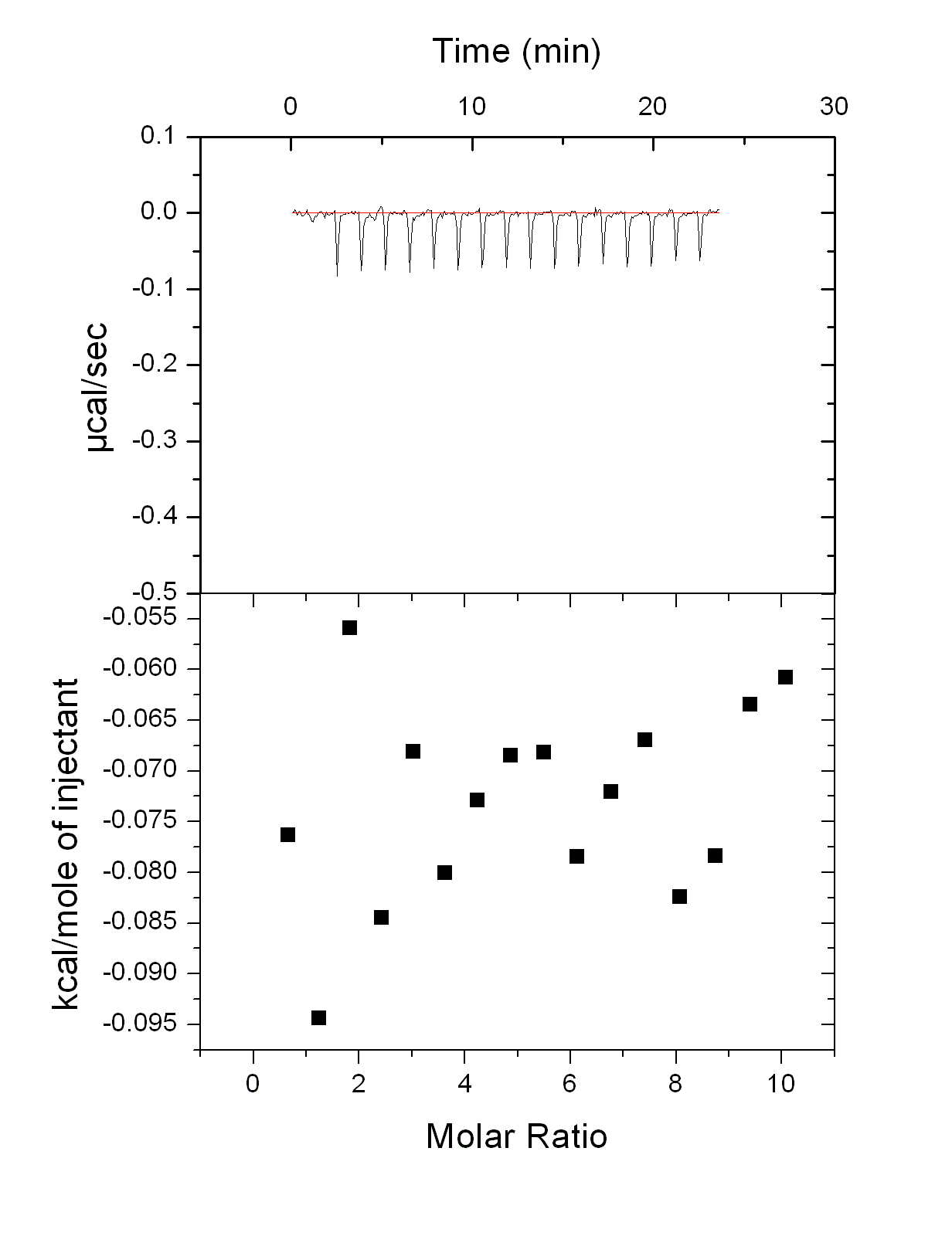

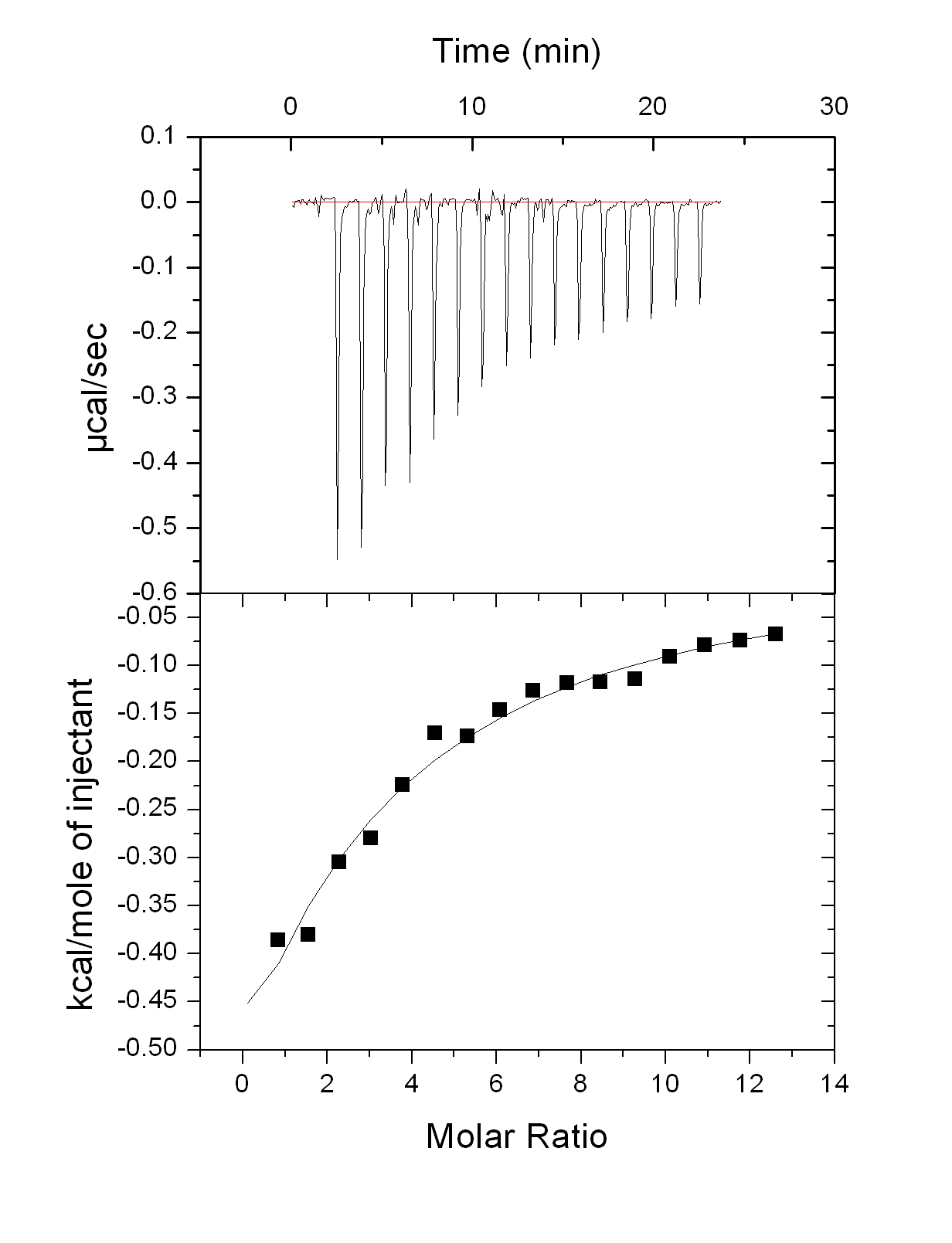

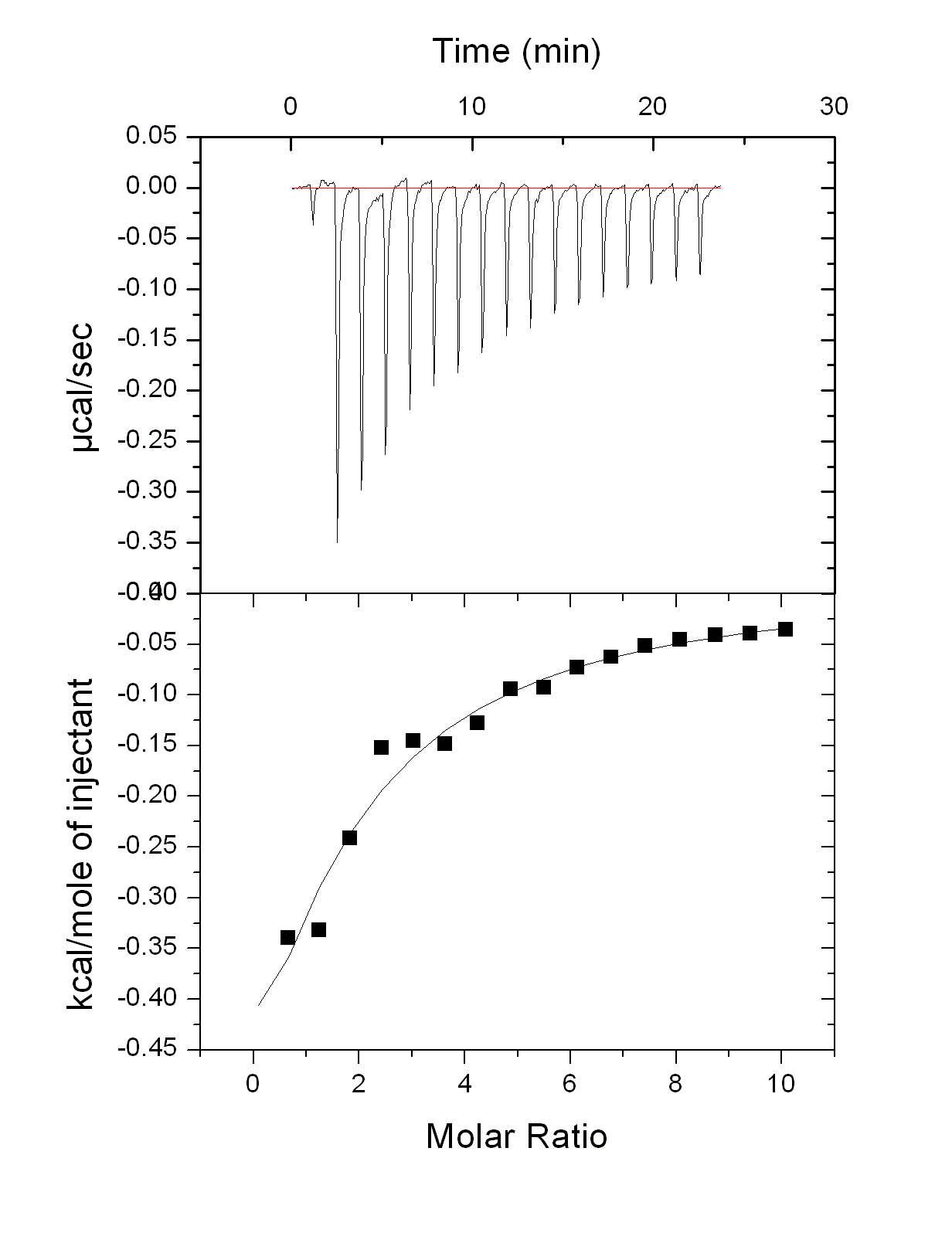

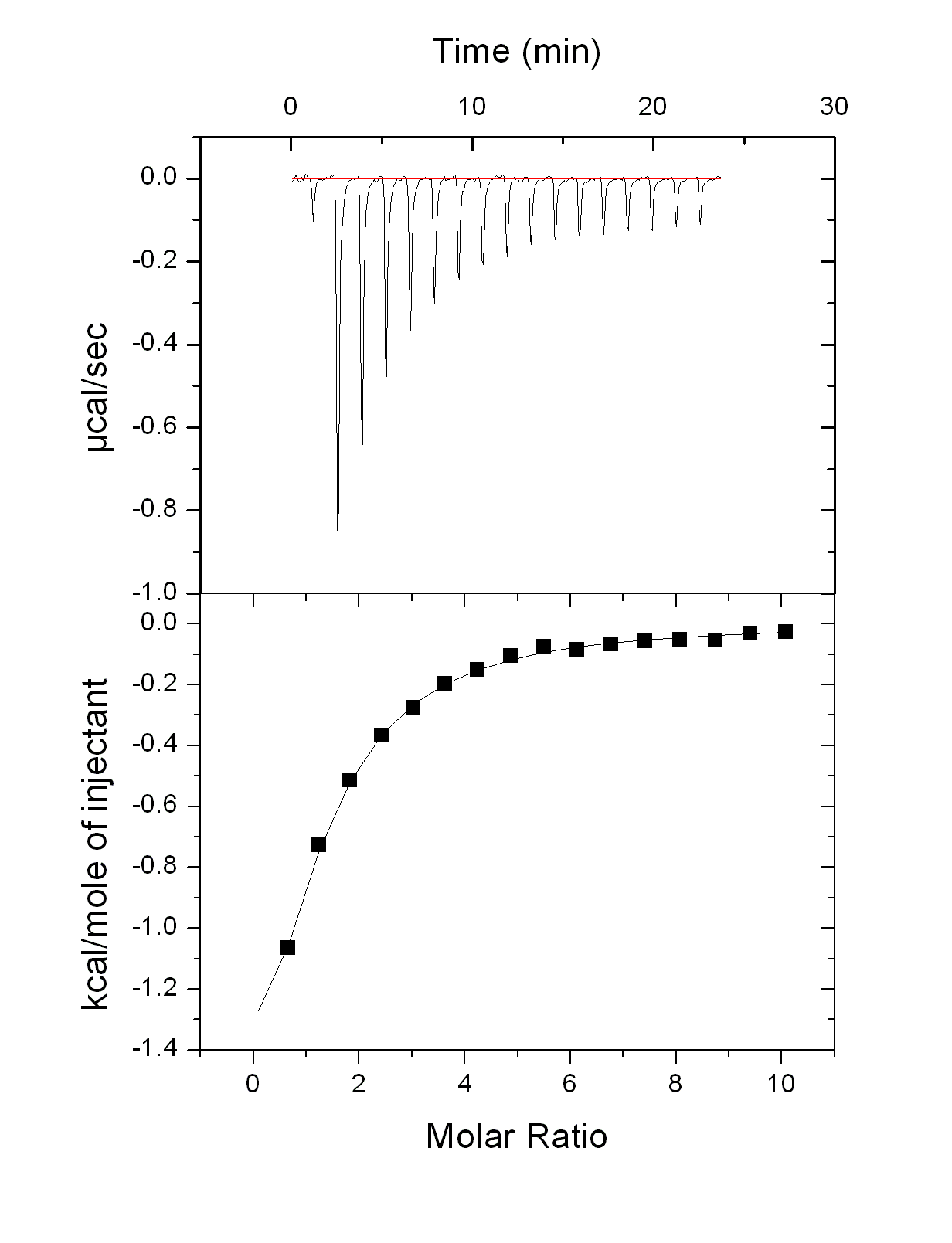

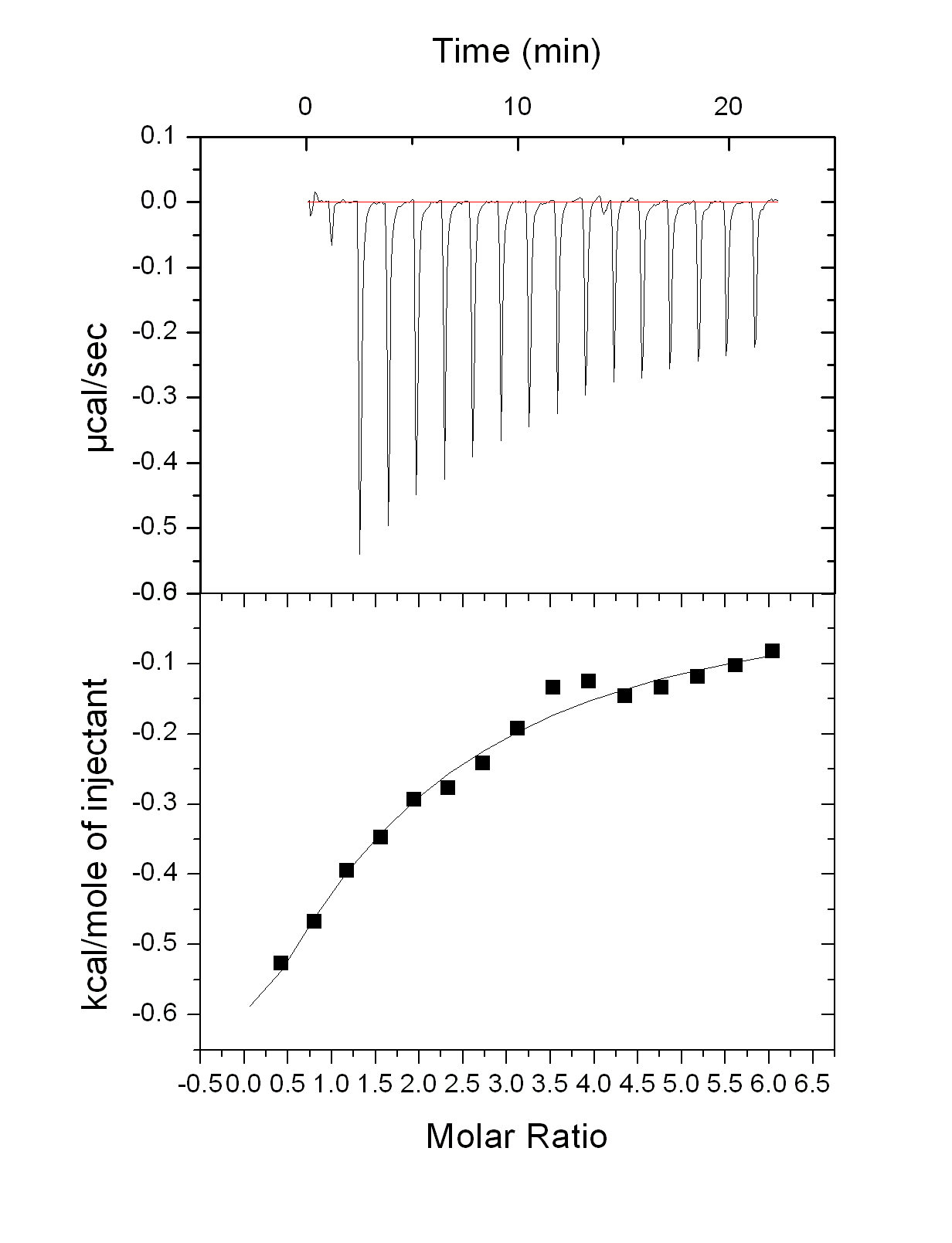


WHTA

FATA

FHAA

FHTG

FHTU

FNTA

FPTA

FHPA

FHTA

###
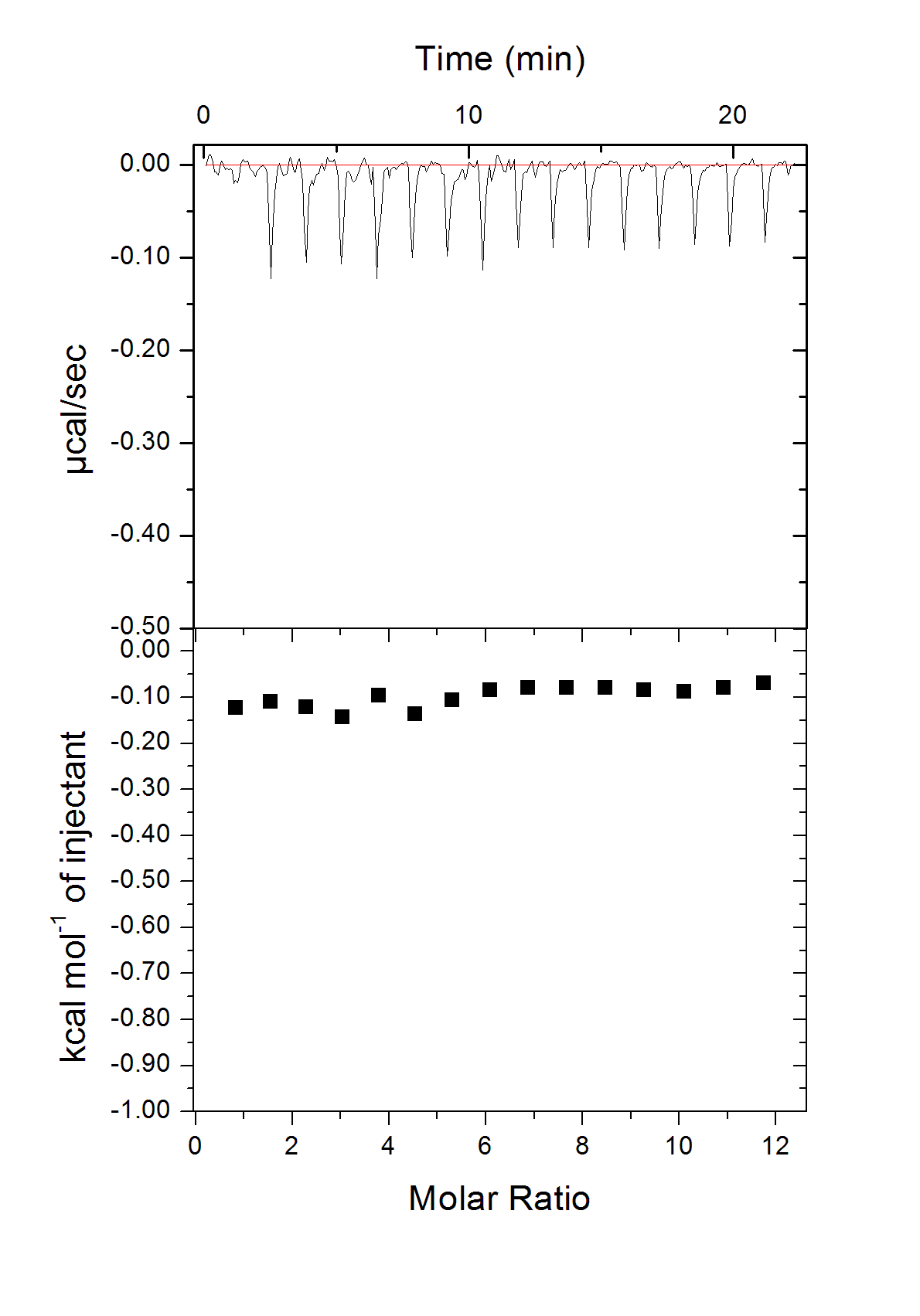

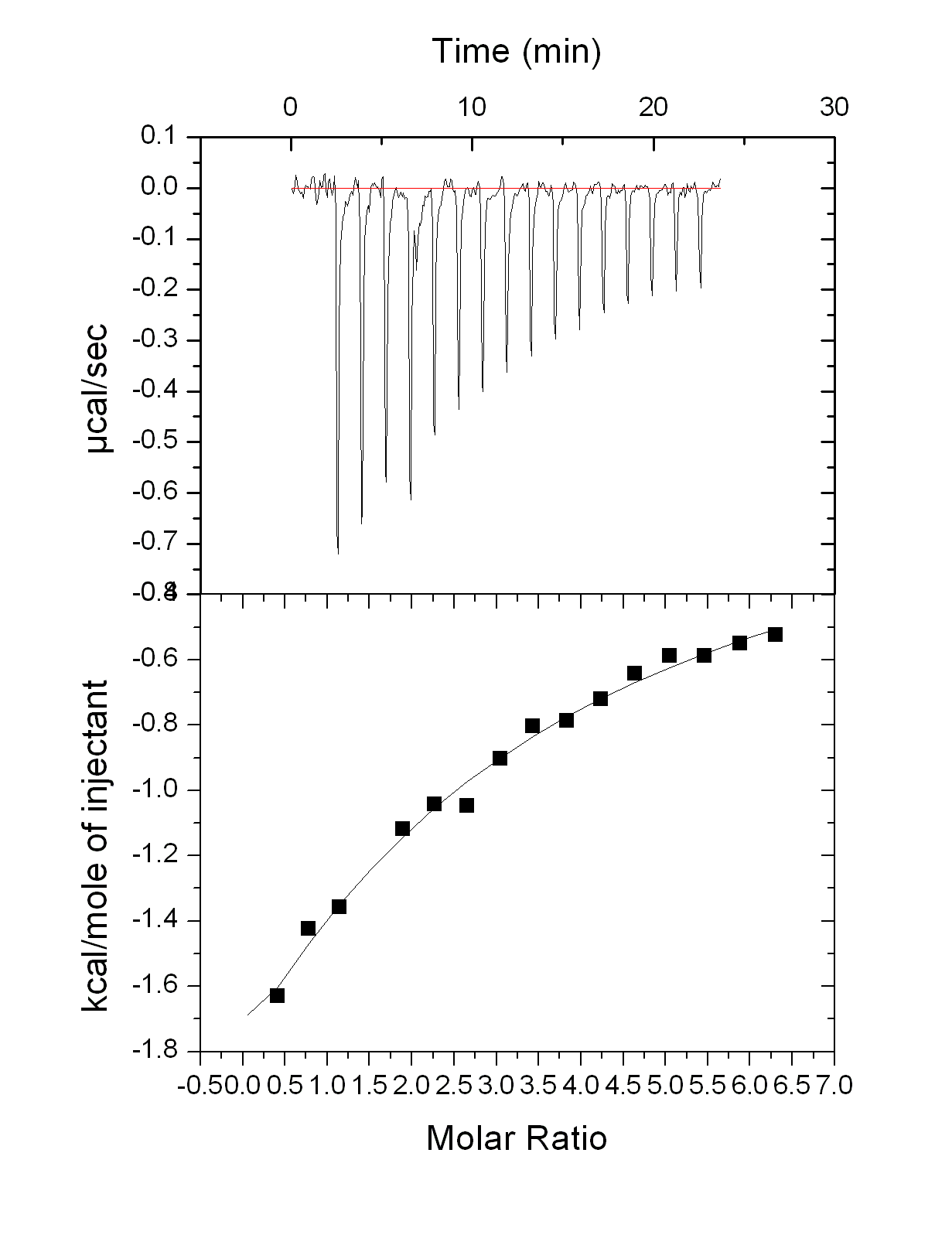

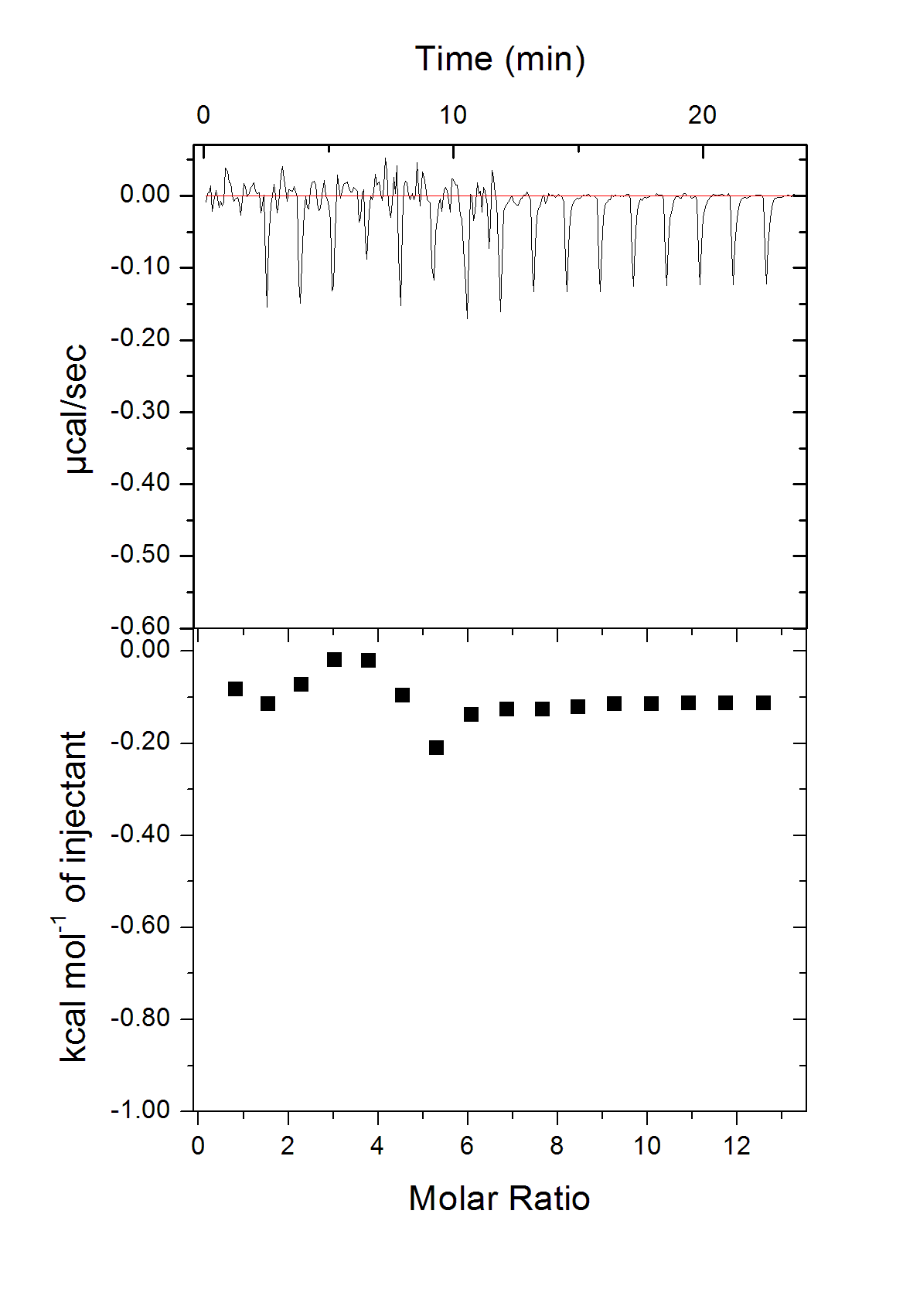
Supplementary Figure 2: ITC data for Table 2 entries 10-13

FH

WHPA


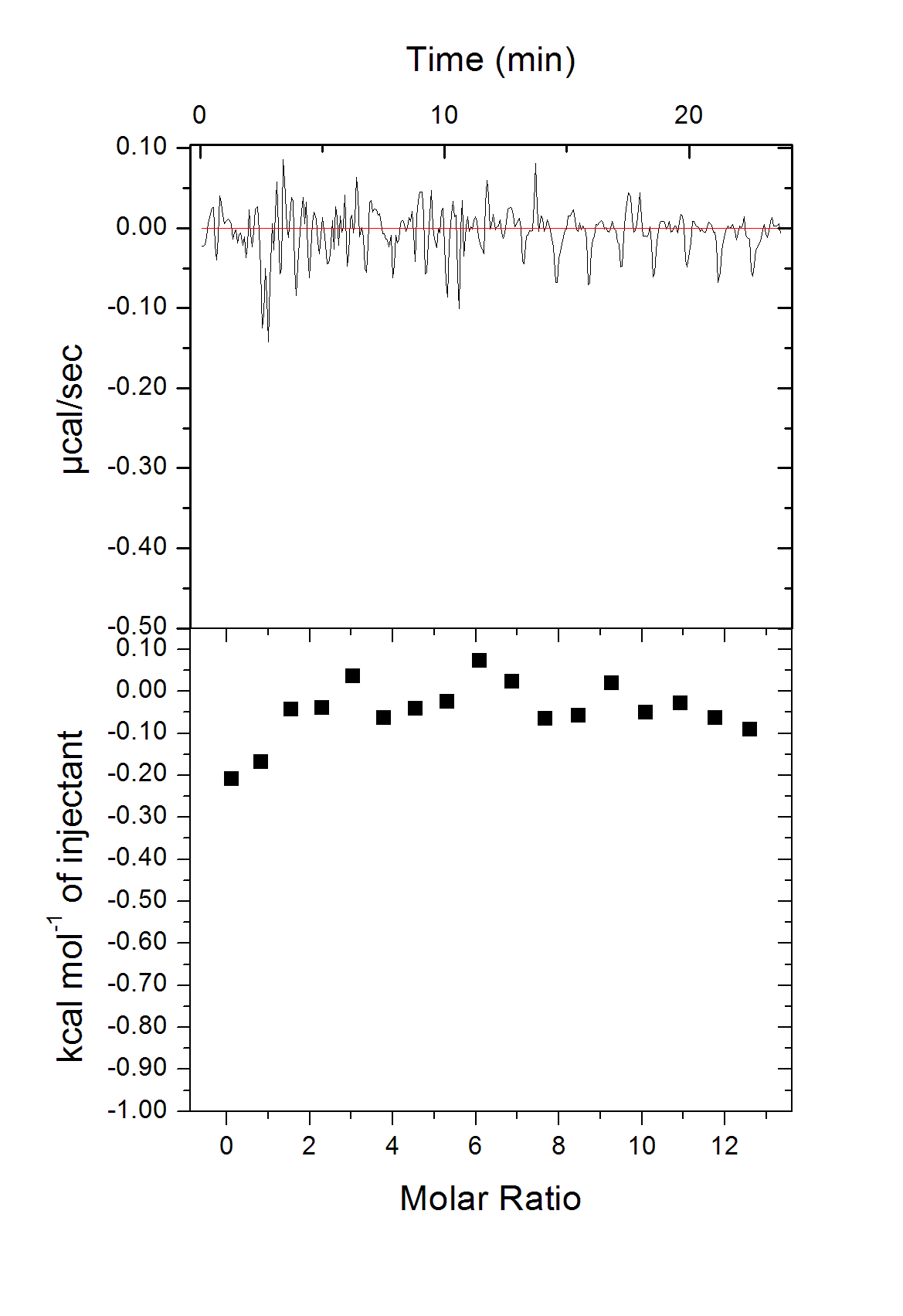


FHT

HTA

### Supplementary table 1. Crystallographic data collection, refinement and structure analysis

|  | **HumRadA1 +**  **WHTA peptide** | **HumRadA1 +**  **FHAA peptide** | **HumRadA1 +**  **FHPA peptide** | **HumRadA1 +**  **FHTG peptide** | **HumRadA1 +**  **FHTU peptide** | **RadAct +**  **FATA peptide** |
| --- | --- | --- | --- | --- | --- | --- |
|  | PDB: 5FOW | PDB: 5FOX | PDB: 5FOU | PDB: 5FOV | PDB: 5FOT | PDB: 5FPK |
| **Data collection and processing:** |  |  |  |  |  |  |
| Synchrotron/ beamline: | Diamond / I03 | Diamond / I02 | Diamond / I04 | SLS / PXIII | SLS / PXIII | ESRF ID 14.4 |
| X-ray wavelength (Å) | 0.9795 | 0.9795 | 0.9795 | 0.9793 | 0.9794 | 0.9795 |
| Resolution limits (high res. shell) (Å) | 33.23-1.80 (1.91 - 1.80) | 36.64-1.30 (1.38-1.30) | 43.96- 1.50 (1.59 – 1.50) | 43.61-1.73 (1.84 - 1.73) | 1.19 (1.26 - 1.19) | 49.8-1.34 (1.42 - 1.34) |
| Completeness (%) | 95.7 (93.5) | 99.8 (99.7) | 99.8 (99.3) | 96.2 (83.9) | 90.8 (65.1) | 98.3 (92.3) |
| Number of unique reflections | 38505(6051) | 55533 (8849) | 35168(5563) | 42203(5928) | 63089(7206) | 47749(7144) |
| Multiplicity | 2.8 (2.8) | 6.7 (6.2) | 7.0 (6.7) | 3.6 (3.1) | 3.2 (1.8) | 7.0 (6.2) |
| R_merge_ | 0.081 (0.339) | 0.094 (0.807) | 0.125 (0.863) | 0.120 (0.457) | 0.048 (0.268) | 0.069 (0.608) |
| R_meas_ | 0.100 (0.422) | 0.102 (0.881) | 0.135 (0.936) | 0.141 (0.548) | 0.056 (0.347) | 0.074 (0.663) |
| I/σ(I) | 9.96 (3.05) | 15.60 (2.44) | 13.96 (2.22) | 10.07 (2.75) | 14.46 (2.76) | 22.41 (2.98) |
| Spacegroup | P2_1_ | P2_1_2_1_2 | P2_1_2_1_2_1_ | P2_1_ | P2_1_2_1_2_1_ | P2_1_2_1_2_1_ |
| Unit cell (a, b, c ) (Å) | 40.5, 87.6, 61.9 | 46.8, 117.6, 40.3 | 40.3, 60.6, 87.9 | 40.2, 87.0, 61.4 | 40.2, 60.6, 87.3 | 40.3, 60.6, 87.5 |
| Unit cell (α, β, γ) (°) | 90.0, 92.4, 90.0 | 90.0, 90.0, 90.0 | 90.0, 90.0, 90.0 | 90.0, 92.3, 90.0, | 90.0, 90.0, 90.0 | 90.0, 90.0, 90.0 |
| Molecules in the ASU | 2 | 1 | 1 | 2 | 1 | 1 |
| **Refinement** |  |  |  |  |  |  |
| Number of reflections /R_free_ set | 38500 / 1979 | 55532 / 2868 | 35160 / 1768 | 42195 / 2172 | 63320 / 3219 | 47745 / 2377 |
| R_cryst_ / R_free_ | 18.3 / 21.8 | 15.6 / 18.2 | 16.4 / 19.2 | 16.9 / 20.4 | 13.3 / 16.1 | 12.3 / 16.4 |
| Rmsd bonds (Å) /angles (°)/planes (°) | 0.006 / 0.808 / 0.004 | 0.006 / 1.139 / 0.005 | 0.007 / 0.958 / 0.046 | 0.006 / 0.786 / 0.005 | 0.010 / 1.154 / 0.007 | 0.09/1.033/0.006 |
| Ramachandran analysis: |  |  |  |  |  |  |
| Most favoured/allowed/outliers (%) | 99.5 0 / 0.5 / 5 | 99.2 / 0.8 / 0.0 | 99.6 / 0.4 / 0.0 | 99.3 / 0.4 / 0.2 | 99.6 / 0.4 / 0.0 | 98.7 / 1.3 / 0.0 |
| Number of non-H atoms: |  |  |  |  |  |  |
| Protein and peptide / solvent atoms | 3546 / 553 | 1974 / 299 | 1883 / 410 | 3645 / 644 | 1977 / 351 | 1891 / 435 |
| Average B factors (Å^2^): |  |  |  |  |  |  |
| Protein / Peptide(s)/ Solvent | 15.9 / 16. 2 / 29.8 | 14.5 / 11.3 / 30.2 | 12.7 / 13.9 / 28.7 | 13.5 / 20.2 /26.7 | 13.6 / 14.2 /27.8 | 12.8/ 20.5 / 31.2 |

### Supplementary Figure 3. Sequence alignments used for consensus diagram in Figure 1C for BRC repeats 1,2,3,4,6,7 and 8, along with consensus diagrams for each individual BRC repeat.

### BRC1:

ENSAMXP00000013440_Amex/1-2700 DSGFKTASNR

ENSLAFP00000002234_Lafr/1-3302 GGGFRTASDK

ENSMICP00000010933_Mmur/1-3176 GGSFRTASNK

ENSPPYP00000005997_Pabe/1-3415 GGGFRTASNK

ENSPFOP00000001575_Pfor/1-3036 SEAFRTAGGN

ENSPVAP00000000225_Pvam/1-3376 GNGFRTASNK

ENSOGAP00000009477_Ogar/1-3406 GGGFRTASNK

ENSOPRP00000014082_Opri/1-3185 GSSFRTASNK

ENSPTRP00000009812_Ptro/1-3418 GGSFRTASNK

ENSOCUP00000014514_Ocun/1-3400 GGSFRTASNK

ENSTSYP00000000441_Tsyr/1-3416 GGSFRTASNK

ENSVPAP00000000821_Vpac/1-3392 GNGFRTASNK

ENSCSAP00000013938_Csab/1-3420 GGSFRTASNK

ENSTGUP00000012130_Tgut/1-3305 FSGFQTASNK

ENSGGOP00000015446_Ggor/1-3333 GGSFRTASNK

ENSMLUP00000012516_Mluc/1-3376 GHGFRTASNK

ENSXETP00000060681_Xtro/1-3202 FKGFKTASNK

ENSMODP00000033276_Mdom/1-3335 GCGFKTASNK

ENSAPLP00000007411_Apla/1-3394 FGGFQTASNK

ENSGALP00000027524_Ggal/1-3397 FGGFQTASNK

ENSFALP00000008821_Falb/1-3362 FSGFQTASNK

ENSECAP00000013146_Ecab/1-3419 GHGFRTASNK


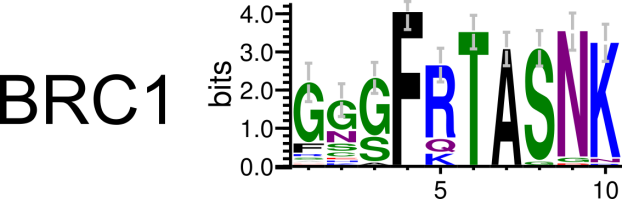
ENSCHOP00000007822_Chof/1-3436 GCGFRTASNK

ENSDORP00000006609_Dord/1-3367 RGGFRTASNK

ENSSHAP00000012162_Shar/1-3315 GSGFKTASNK

ENSMPUP00000001928_Mpfu/1-3466 GNGFRTASNK

ENSSTOP00000004979_Itri/1-3363 GGSFRTASNK

ENSCPOP00000004635_Cpor/1-3313 GGGFRTASNK

ENSCAFP00000009557_Cfam/1-3446 ENGFKTASNK

ENSXMAP00000006983_Xmac/1-3052 SEAFRTAGGN

ENSFCAP00000019777_Fcat/1-3360 GNGFRTASNK

ENSCJAP00000034250_Cjac/1-3409 GGSFRTASNK

ENSSARP00000002541_Sara/1-3357 GKGFRTASNK

ENSBTAP00000001311_Btau/1-3357 GNGFRTASNK

ENSOANP00000024376_Oana/1-3459 GCGFQTASNK

ENSOARP00000011988_Oari/1-3427 GNGFRTASNK

ENSTBEP00000013856_Tbel/1-3177 GGSFRTASNK

ENSNLEP00000001277_Nleu/1-3419 GGSFRTASNK

ENSMGAP00000015990_Mgal/1-3387 FGGFQTASNK

ENSETEP00000003277_Etel/1-3335 GGGFRTASNK

ENSDNOP00000034947_Dnov/1-3450 GCGFRTASNK

ENSMUSP00000038576_Mmus/1-3329 GGSFRTASNK

ENSRNOP00000001475_Rnor/1-3343 GGSFRTASNK

ENSONIP00000006940_Onil/1-2832 ASGFKTASNK

ENSPSIP00000012858_Psin/1-3083 G-GFQTASNK

ENSP00000369497_Hsap/1-3418 GGSFRTASNK

ENSEEUP00000008968_Eeur/1-3345 GNGFRTASNK

ENSAMEP00000009909_Amel/1-3460 RNGFRTASNK

### BRC2:

ENSAMXP00000013440_Amex/1-2700 CVGFKTARGN

ENSLAFP00000002234_Lafr/1-3302 FTGFCSALGT

ENSMICP00000010933_Mmur/1-3176 FRGFYSALGK

ENSPPYP00000005997_Pabe/1-3415 FRGFYSAHGA

ENSPFOP00000001575_Pfor/1-3036 GDGFCTAAGK

ENSPVAP00000000225_Pvam/1-3376 FRGFYSAHGT

ENSOGAP00000009477_Ogar/1-3406 FRGFYSARGK

ENSPTRP00000009812_Ptro/1-3418 FRGFYSAHGT

ENSOCUP00000014514_Ocun/1-3400 FRGFYSALGT

ENSMMUP00000009432_Mmul/1-3336 FRGFYSAHGV

ENSTSYP00000000441_Tsyr/1-3416 FRGFYSARGK

ENSVPAP00000000821_Vpac/1-3392 FRGFYSARGT

ENSCSAP00000013938_Csab/1-3420 FRGFYSAHGV

ENSTGUP00000012130_Tgut/1-3305 DESFPIAKQD

ENSGGOP00000015446_Ggor/1-3333 FRGFYSAHGA

ENSMLUP00000012516_Mluc/1-3376 FRGFYSARGT

ENSXETP00000060681_Xtro/1-3202 FAGFNLASGK

ENSMODP00000033276_Mdom/1-3335 FKGFCSALGR

ENSAPLP00000007411_Apla/1-3394 FVGFTSAGGK

ENSGALP00000027524_Ggal/1-3397 FVGFTSAGGK

ENSFALP00000008821_Falb/1-3362 FIGFTSAGGK

ENSECAP00000013146_Ecab/1-3419 FRGFYSARGT

ENSCHOP00000007822_Chof/1-3436 FKGFYSALGT

ENSDORP00000006609_Dord/1-3367 FQGFYSALGT

ENSSHAP00000012162_Shar/1-3315 FKGFCSALGR

ENSMPUP00000001928_Mpfu/1-3466 FRGFYSARGT


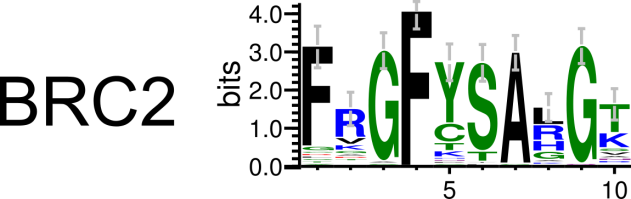
ENSSTOP00000004979_Itri/1-3363 FRGFYSARGT

ENSCPOP00000004635_Cpor/1-3313 YRGFYSGLGT

ENSCAFP00000009557_Cfam/1-3446 FRGFYSARGT

ENSDARP00000099674_Drer/1-2874 TFGFKTAKGK

ENSXMAP00000006983_Xmac/1-3052 GDGFCTAAGK

ENSFCAP00000019777_Fcat/1-3360 FRGFYSARGT

ENSCJAP00000034250_Cjac/1-3409 LRGFYSAHGV

ENSSARP00000002541_Sara/1-3357 FKGFYSALGS

ENSBTAP00000001311_Btau/1-3357 FRGFYSARGT

ENSOANP00000024376_Oana/1-3459 FRGFHSALGS

ENSOARP00000011988_Oari/1-3427 FKGFYSARGT

ENSNLEP00000001277_Nleu/1-3419 FRGFYSAHGS

ENSMGAP00000015990_Mgal/1-3387 FVGFTSAGGK

ENSETEP00000003277_Etel/1-3335 FTGFCSALGT

ENSDNOP00000034947_Dnov/1-3450 FGGFYSALGT

ENSMUSP00000038576_Mmus/1-3329 FGGFCSALGT

ENSRNOP00000001475_Rnor/1-3343 FGGFCSALGT

ENSONIP00000006940_Onil/1-2832 SVAFKTAGGN

ENSPSIP00000012858_Psin/1-3083 FAGFSSAGGK

ENSPCAP00000000440_Pcap/1-3374 FVGFCSAGGT

ENSP00000369497_Hsap/1-3418 FRGFYSAHGT

ENSEEUP00000008968_Eeur/1-3345 FRGFYSALGT

ENSAMEP00000009909_Amel/1-3460 FRGFYSARGI

### BRC3:

ENSMICP00000010933_Mmur/1-3176 DISFQTASGK

ENSPPYP00000005997_Pabe/1-3415 DTSFQTASGK

ENSPFOP00000001575_Pfor/1-3036 CSGFTTAGGA

ENSPVAP00000000225_Pvam/1-3376 DISFQTASGK

ENSOGAP00000009477_Ogar/1-3406 DISFQTASGK

ENSPTRP00000009812_Ptro/1-3418 DTFFQTASGK

ENSOCUP00000014514_Ocun/1-3400 DISFQTAAGR

ENSMMUP00000009432_Mmul/1-3336 DISFQTASGK

ENSTSYP00000000441_Tsyr/1-3416 DISFHTASGK

ENSVPAP00000000821_Vpac/1-3392 GLSFQTASGK

ENSCSAP00000013938_Csab/1-3420 GISFQTASGK

ENSGGOP00000015446_Ggor/1-3333 DTFFQTASGK

ENSMLUP00000012516_Mluc/1-3376 DISFKTASGK

ENSXETP00000060681_Xtro/1-3202 MKGFQTASGR

ENSMODP00000033276_Mdom/1-3335 LQSFQTASGR

ENSAPLP00000007411_Apla/1-3394 LPQKFQTSDG

ENSECAP00000013146_Ecab/1-3419 DLSFQTASGK

ENSCHOP00000007822_Chof/1-3436 YLPFQTASGK


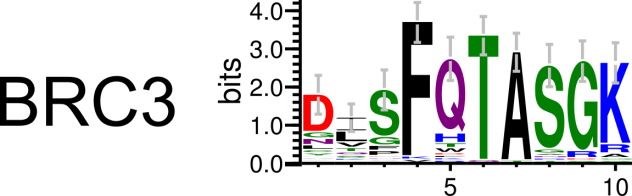
ENSSHAP00000012162_Shar/1-3315 LQHFQTASGR

ENSMPUP00000001928_Mpfu/1-3466 DLSFQTASGK

ENSSTOP00000004979_Itri/1-3363 DISFQTASGK

ENSCPOP00000004635_Cpor/1-3313 GISFQTASGK

ENSCAFP00000009557_Cfam/1-3446 YLSFQTASRK

ENSDARP00000099674_Drer/1-2874 GYGFQTASGK

ENSXMAP00000006983_Xmac/1-3052 CSGFTTAGGA

ENSFCAP00000019777_Fcat/1-3360 DVSFQTASGK

ENSCJAP00000034250_Cjac/1-3409 DLSFQTASGK

ENSSARP00000002541_Sara/1-3357 DVYFQTASGK

ENSBTAP00000001311_Btau/1-3357 DLSFWTANGK

ENSOANP00000024376_Oana/1-3459 PQSFQTARGK

ENSOARP00000011988_Oari/1-3427 DLSFWTASRK

ENSTBEP00000013856_Tbel/1-3177 DISFQTASGK

ENSNLEP00000001277_Nleu/1-3419 DKSFQTASGK

ENSETEP00000003277_Etel/1-3335 NVPFQTASGK

ENSMUSP00000038576_Mmus/1-3329 NISFQTASGK

ENSRNOP00000001475_Rnor/1-3343 NISFQTASGK

ENSONIP00000006940_Onil/1-2832 TSGFDTATGK

ENSPSIP00000012858_Psin/1-3083 NVDFRTASSS

ENSPCAP00000000440_Pcap/1-3374 DLPVHTASRK

ENSP00000369497_Hsap/1-3418 DTFFQTASGK

ENSEEUP00000008968_Eeur/1-3345 HLSFHTASGK

ENSAMEP00000009909_Amel/1-3460 DVSFHTASGK

### BRC4:

ENSAMXP00000013440_Amex/1-2700 SSGFKMASGK

ENSLAFP00000002234_Lafr/1-3302 MLGFHTASGK

ENSMICP00000010933_Mmur/1-3176 LLSFHTASGK

ENSPPYP00000005997_Pabe/1-3415 LLGFHTASGK

ENSPFOP00000001575_Pfor/1-3036 SCGFKTASGK

ENSPVAP00000000225_Pvam/1-3376 MLDFHTANGK

ENSOGAP00000009477_Ogar/1-3406 LLSFHTASGK

ENSPTRP00000009812_Ptro/1-3418 LLGFHTASGK

ENSOCUP00000014514_Ocun/1-3400 LLGFHTASGK

ENSMMUP00000009432_Mmul/1-3336 LLGFHTASGK

ENSTSYP00000000441_Tsyr/1-3416 LWSFHTASGR

ENSVPAP00000000821_Vpac/1-3392 MLGFHTASGK

ENSCSAP00000013938_Csab/1-3420 LLGFHTASGK

ENSTGUP00000012130_Tgut/1-3305 LTGFRTASGK

ENSGGOP00000015446_Ggor/1-3333 LLGFHTASGK

ENSMLUP00000012516_Mluc/1-3376 MLGFHTASGK

ENSMODP00000033276_Mdom/1-3335 MVGFHTASGK

ENSAPLP00000007411_Apla/1-3394 LTGFQTASGK

ENSGALP00000027524_Ggal/1-3397 LTGFCTASGK


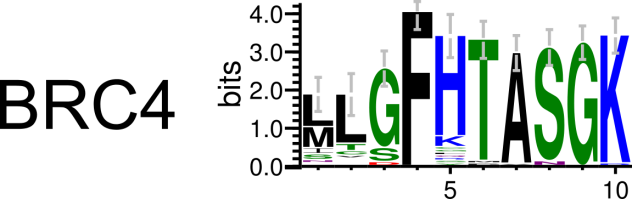
ENSFALP00000008821_Falb/1-3362 LTGFHTASGK

ENSECAP00000013146_Ecab/1-3419 MLGFHTASGK

ENSCHOP00000007822_Chof/1-3436 MLGFHTASGK

ENSSHAP00000012162_Shar/1-3315 MVGFHTASGK

ENSMPUP00000001928_Mpfu/1-3466 MLGFHTASGK

ENSSTOP00000004979_Itri/1-3363 LLSFHTASGK

ENSCPOP00000004635_Cpor/1-3313 LLGFYTASGK

ENSCAFP00000009557_Cfam/1-3446 VLGFHTASGK

ENSDARP00000099674_Drer/1-2874 NCGFSTASGK

ENSXMAP00000006983_Xmac/1-3052 SCGFKTASGK

ENSFCAP00000019777_Fcat/1-3360 ILGFHTASGK

ENSCJAP00000034250_Cjac/1-3409 LLGFHTASGK

ENSSARP00000002541_Sara/1-3357 ILGFHTASGK

ENSBTAP00000001311_Btau/1-3357 TLGFHTASGK

ENSOANP00000024376_Oana/1-3459 IMDFKTASGK

ENSOARP00000011988_Oari/1-3427 MLGFHTASGK

ENSTBEP00000013856_Tbel/1-3177 LLSFLTASGK

ENSNLEP00000001277_Nleu/1-3419 LLGFHTASGK

ENSMGAP00000015990_Mgal/1-3387 LTGFHTASGK

ENSETEP00000003277_Etel/1-3335 MLGFHTASGK

ENSDNOP00000034947_Dnov/1-3450 MLGFHTASGK

ENSMUSP00000038576_Mmus/1-3329 LLSFHTASGK

ENSRNOP00000001475_Rnor/1-3343 LLSFHTASGK

ENSONIP00000006940_Onil/1-2832 SGGFGPVSGK

ENSPSIP00000012858_Psin/1-3083 NNGFHTANGK

ENSPCAP00000000440_Pcap/1-3374 MLGFHTASGK

ENSP00000369497_Hsap/1-3418 LLGFHTASGK

ENSEEUP00000008968_Eeur/1-3345 ILGFHTASGK

ENSAMEP00000009909_Amel/1-3460 MLGFHTASGK

### BRC6:

ENSAMXP00000013440_Amex/1-2700 ANGFKMASGK

ENSAMXP00000013440_Amex/1-2700 GCGFSTASGK

ENSLAFP00000002234_Lafr/1-3302 PPVFSTASGK

ENSMICP00000010933_Mmur/1-3176 PPAFSTASGK

ENSPPYP00000005997_Pabe/1-3415 PPAFRIASGK

ENSPFOP00000001575_Pfor/1-3036 SCGFQTASGK

ENSPVAP00000000225_Pvam/1-3376 SPAFSTAGGQ

ENSOGAP00000009477_Ogar/1-3406 TPAFSTASGK

ENSOPRP00000014082_Opri/1-3185 PPAFSTASGK

ENSPTRP00000009812_Ptro/1-3418 PPAFRIASGK

ENSOCUP00000014514_Ocun/1-3400 PPIFSTASGK

ENSMMUP00000009432_Mmul/1-3336 PPAFSTASGK

ENSVPAP00000000821_Vpac/1-3392 PPAFGTASDK

ENSCSAP00000013938_Csab/1-3420 PPAFSTASGK

ENSTGUP00000012130_Tgut/1-3305 QTVFSTAKGK

ENSGGOP00000015446_Ggor/1-3333 PPAFRIASDE

ENSMLUP00000012516_Mluc/1-3376 SPAFSTASGQ

ENSXETP00000060681_Xtro/1-3202 PLSFSTASGK

ENSMODP00000033276_Mdom/1-3335 PPAFTTASGK


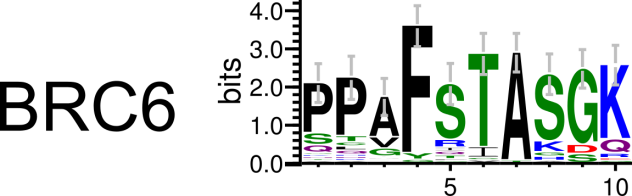
ENSAPLP00000007411_Apla/1-3394 QTVFSTAKGK

ENSGALP00000027524_Ggal/1-3397 KQVFSTAKGK

ENSFALP00000008821_Falb/1-3362 QTVFSTAKGR

ENSECAP00000013146_Ecab/1-3419 VPAFSTASGK

ENSCHOP00000007822_Chof/1-3436 PPAFSTASGK

ENSSHAP00000012162_Shar/1-3315 PPTFTTASGK

ENSSTOP00000004979_Itri/1-3363 PPAFSTASGK

ENSCPOP00000004635_Cpor/1-3313 PPAFSTASSK

ENSDARP00000099674_Drer/1-2874 NVGFSTAGGK

ENSXMAP00000006983_Xmac/1-3052 SRGFQTASGK

ENSCJAP00000034250_Cjac/1-3409 PHAFSTASGK

ENSBTAP00000001311_Btau/1-3357 PPAYSTASDQ

ENSOARP00000011988_Oari/1-3427 PPAYSTASDQ

ENSTBEP00000013856_Tbel/1-3177 PPAFSTASGK

ENSNLEP00000001277_Nleu/1-3419 PPAFSTASGK

ENSMGAP00000015990_Mgal/1-3387 -QMFSTAKGK

ENSETEP00000003277_Etel/1-3335 PPAFSTASGK

ENSDNOP00000034947_Dnov/1-3450 PPAFSTASGK

ENSMUSP00000038576_Mmus/1-3329 SLVFITAHSQ

ENSRNOP00000001475_Rnor/1-3343 SPVFITTHSQ

ENSONIP00000006940_Onil/1-2832 QNGGFCAASG

ENSPCAP00000000440_Pcap/1-3374 PPAFSVASGR

ENSP00000369497_Hsap/1-3418 PPAFRIASGK

### BRC7:

ENSAMXP00000013440_Amex/1-2700 CLGFSTASGK

ENSLAFP00000002234_Lafr/1-3302 CGIFSTASGK

ENSMICP00000010933_Mmur/1-3176 CGIFSTASGK

ENSPPYP00000005997_Pabe/1-3415 CGIFSTASGK

ENSPFOP00000001575_Pfor/1-3036 CCGFTTAKGE

ENSPVAP00000000225_Pvam/1-3376 CGVFSTAGGK

ENSOGAP00000009477_Ogar/1-3406 -EIFSTASGK

ENSOPRP00000014082_Opri/1-3185 SGIFSTANGK

ENSPTRP00000009812_Ptro/1-3418 CGIFSTASGK

ENSOCUP00000014514_Ocun/1-3400 -GVFSTASGK

ENSMMUP00000009432_Mmul/1-3336 CGIFSTASGK

ENSVPAP00000000821_Vpac/1-3392 CGVFSTASGK

ENSCSAP00000013938_Csab/1-3420 CGIFSTASGK

ENSTGUP00000012130_Tgut/1-3305 FGFFSTASGK

ENSGGOP00000015446_Ggor/1-3333 CGIFSTASGK

ENSMLUP00000012516_Mluc/1-3376 CGIFSTASGK

ENSXETP00000060681_Xtro/1-3202 AVSFSTASGK

ENSMODP00000033276_Mdom/1-3335 VGIFCTANGK

ENSAPLP00000007411_Apla/1-3394 LGFFSTASGK

ENSGALP00000027524_Ggal/1-3397 LGFFSTASGK

ENSFALP00000008821_Falb/1-3362 FGFFNTASGK

ENSECAP00000013146_Ecab/1-3419 CGIFSTASGK

ENSCHOP00000007822_Chof/1-3436 CGIFSTASGK

ENSSHAP00000012162_Shar/1-3315 VGIFCTANGK

ENSMPUP00000001928_Mpfu/1-3466 CGIFSTASGK

ENSSTOP00000004979_Itri/1-3363 CGIFSTASGK


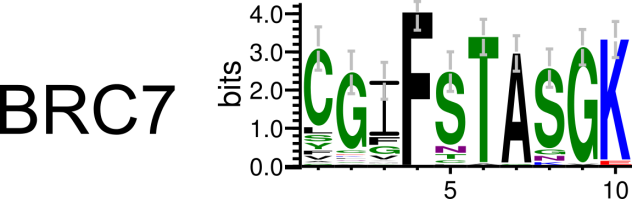
ENSCPOP00000004635_Cpor/1-3313 YGIFSTASGK

ENSCAFP00000009557_Cfam/1-3446 CGIFSTASGK

ENSDARP00000099674_Drer/1-2874 CKGFTTASGK

ENSXMAP00000006983_Xmac/1-3052 CCGFTTAKGE

ENSFCAP00000019777_Fcat/1-3360 CGIFSTASGK

ENSCJAP00000034250_Cjac/1-3409 CGIFSTASGK

ENSSARP00000002541_Sara/1-3357 CGIFSTASGK

ENSBTAP00000001311_Btau/1-3357 CGIFNTASGK

ENSOANP00000024376_Oana/1-3459 SQAFNTASGK

ENSOARP00000011988_Oari/1-3427 CGIFNTASGK

ENSTBEP00000013856_Tbel/1-3177 CGIFSTASGK

ENSNLEP00000001277_Nleu/1-3419 CGMFSTGSGK

ENSMGAP00000015990_Mgal/1-3387 LGFFSTASGK

ENSDNOP00000034947_Dnov/1-3450 CGIFSTASGK

ENSMUSP00000038576_Mmus/1-3329 YGIFSTASGK

ENSRNOP00000001475_Rnor/1-3343 YGIFSTASGK

ENSONIP00000006940_Onil/1-2832 SSGFLAAGVK

ENSPCAP00000000440_Pcap/1-3374 CGIFSTAGGK

ENSP00000369497_Hsap/1-3418 CGIFSTASGK

ENSEEUP00000008968_Eeur/1-3345 GGIFSTASGK

ENSAMEP00000009909_Amel/1-3460 CGIFSTASGK

### BRC8:

ENSAMXP00000013440_Amex/1-2700 QFGFSTASGK

ENSPPYP00000005997_Pabe/1-3415 FSGFSTASGK

ENSPVAP00000000225_Pvam/1-3376 FSGFSTASGK

ENSOGAP00000009477_Ogar/1-3406 FSGFSTASGK

ENSOPRP00000014082_Opri/1-3185 FSGFSTASGK

ENSPTRP00000009812_Ptro/1-3418 FSGFSTASGK

ENSOCUP00000014514_Ocun/1-3400 FSGFSTASGK

ENSMMUP00000009432_Mmul/1-3336 FSGFSTASGK

ENSVPAP00000000821_Vpac/1-3392 FSGFSTASGK

ENSCSAP00000013938_Csab/1-3420 FSGFSTASGK

ENSTGUP00000012130_Tgut/1-3305 AFGFSTASGK

ENSGGOP00000015446_Ggor/1-3333 FSGFSTASGK

ENSMLUP00000012516_Mluc/1-3376 FSGFSTASGK

ENSXETP00000060681_Xtro/1-3202 TFGFNTASGK

ENSMODP00000033276_Mdom/1-3335 NSGFNTASGK

ENSAPLP00000007411_Apla/1-3394 AFGFSTASGK

ENSGALP00000027524_Ggal/1-3397 ALGFSTASGK

ENSFALP00000008821_Falb/1-3362 AFGFSTASGK

ENSECAP00000013146_Ecab/1-3419 FSGFSTASGK

ENSCHOP00000007822_Chof/1-3436 YSGFNTASGK

ENSSHAP00000012162_Shar/1-3315 NSGFSTASGK


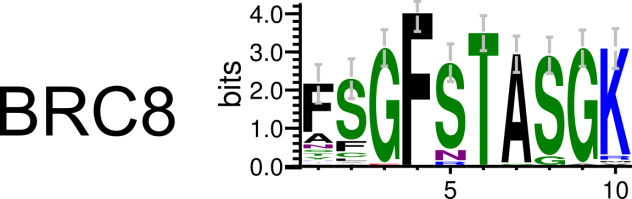
ENSMPUP00000001928_Mpfu/1-3466 FSGFSTASGK

ENSSTOP00000004979_Itri/1-3363 FSGFSTASGK

ENSCPOP00000004635_Cpor/1-3313 FSGFSTASGK

ENSCAFP00000009557_Cfam/1-3446 FSGFRTASGK

ENSDARP00000099674_Drer/1-2874 NIGFSTAGGK

ENSFCAP00000019777_Fcat/1-3360 FSGFSTASGK

ENSCJAP00000034250_Cjac/1-3409 FSGFNTASGK

ENSSARP00000002541_Sara/1-3357 FSGFSTASGR

ENSBTAP00000001311_Btau/1-3357 FSGFSTASGK

ENSOANP00000024376_Oana/1-3459 SSGFRTGSGK

ENSOARP00000011988_Oari/1-3427 FSGFSTASGK

ENSTBEP00000013856_Tbel/1-3177 FCGFSTASGK

ENSNLEP00000001277_Nleu/1-3419 FSGFSTASGR

ENSMGAP00000015990_Mgal/1-3387 ALGFSTASGK

ENSETEP00000003277_Etel/1-3335 AAGFSTASAM

ENSDNOP00000034947_Dnov/1-3450 YSEFSTASGK

ENSMUSP00000038576_Mmus/1-3329 FSGFSTAGGK

ENSRNOP00000001475_Rnor/1-3343 FSGFSTAGGK

ENSONIP00000006940_Onil/1-2832 HCGFSTAGGV

ENSPSIP00000012858_Psin/1-3083 TFGFNTASGK

ENSPCAP00000000440_Pcap/1-3374 SSGFSTASGK

ENSP00000369497_Hsap/1-3418 FSGFSTASGK

ENSEEUP00000008968_Eeur/1-3345 ICGFSTASGK

ENSAMEP00000009909_Amel/1-3460 FSGFSTASGK

### Supplementary figure 4. BRC5 sequences and consensus aligment

### BRC5:

ENSAMXP00000013440_Amex/1-2700 ANGFKMASGK

ENSLAFP00000002234_Lafr/1-3302 ALAFYTGRGR

ENSMICP00000010933_Mmur/1-3176 ALAFYTGHGR

ENSPPYP00000005997_Pabe/1-3415 ALAFYTSCSR

ENSPFOP00000001575_Pfor/1-3036 SCGFTTARGE

ENSPVAP00000000225_Pvam/1-3376 VLAFYTGHGR

ENSOGAP00000009477_Ogar/1-3406 ALAFYTGHGR

ENSPTRP00000009812_Ptro/1-3418 ALAFYTSCSR

ENSOCUP00000014514_Ocun/1-3400 DLAFYTGHGK

ENSMMUP00000009432_Mmul/1-3336 ALAFYTSCSR

ENSTSYP00000000441_Tsyr/1-3416 ALGFHTGHGR

ENSVPAP00000000821_Vpac/1-3392 ALAFYTGHGR

ENSCSAP00000013938_Csab/1-3420 ALAFYTSCSR

ENSTGUP00000012130_Tgut/1-3305 ELESLKGQKI

ENSGGOP00000015446_Ggor/1-3333 ALAFYTSCSR

ENSMLUP00000012516_Mluc/1-3376 AVAFCTGHGR

ENSXETP00000060681_Xtro/1-3202 NSEFTAGEGI

ENSMODP00000033276_Mdom/1-3335 GLGFYTGHGK

ENSAPLP00000007411_Apla/1-3394 ELESLSEWES

ENSGALP00000027524_Ggal/1-3397 EVESLPVHGN


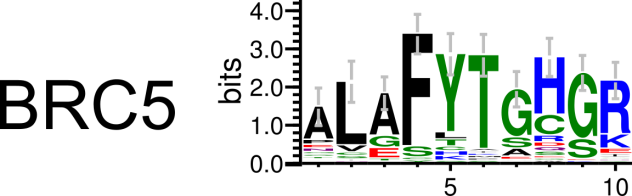
ENSFALP00000008821_Falb/1-3362 ALESHIGQ-K

ENSECAP00000013146_Ecab/1-3419 ALTFYTGCGR

ENSCHOP00000007822_Chof/1-3436 ALAFYTGHGR

ENSSHAP00000012162_Shar/1-3315 GLGFYTGHGK

ENSMPUP00000001928_Mpfu/1-3466 PLAFYTGHGR

ENSSTOP00000004979_Itri/1-3363 ALAFYTGHGK

ENSCPOP00000004635_Cpor/1-3313 PVAFYTGHGK

ENSCAFP00000009557_Cfam/1-3446 PLTFYTGHGR

ENSDARP00000099674_Drer/1-2874 NVGFSTAGGR

ENSXMAP00000006983_Xmac/1-3052 SCGFTTARGE

ENSFCAP00000019777_Fcat/1-3360 ALSFYTGHGR

ENSCJAP00000034250_Cjac/1-3409 ALAFYTYCSR

ENSSARP00000002541_Sara/1-3357 TLAFYTGHGR

ENSBTAP00000001311_Btau/1-3357 ALAFYTGHGR

ENSOANP00000024376_Oana/1-3459 ALEFCTGHGE

ENSOARP00000011988_Oari/1-3427 ALAFYTGHGR

ENSTBEP00000013856_Tbel/1-3177 ALAFYTGHGR

ENSNLEP00000001277_Nleu/1-3419 ALAFYTSCSR

ENSDNOP00000034947_Dnov/1-3450 NLAFYTGHGR

ENSMUSP00000038576_Mmus/1-3329 ALAYYTEDSR

ENSRNOP00000001475_Rnor/1-3343 ALACYTGDSR

ENSONIP00000006940_Onil/1-2832 QSGFKTASGK

ENSPCAP00000000440_Pcap/1-3374 ALEFYTGHGG

ENSP00000369497_Hsap/1-3418 ALAFYTSCSR

ENSEEUP00000008968_Eeur/1-3345 TIAFYTGHGR

ENSAMEP00000009909_Amel/1-3460 PLAFYTGHGR

### Supplementary figure 5. Sequences used in Figure 1D consensus diagram of RAD51/RadA oligomerisation motif

RAD51_BOVIN PMGFTTATEF

RAD51_CANFA PMGFTTATEF

RAD51_RABIT PMGFTTATEF

RAD51_CRIGR PMGFTTATEF

RAD51_HUMAN PMGFTTATEF

RAD51_MOUSE PMGFTTATEF

RAD51_CHICK PMGFTTATEF

G4V6K0_SCHMA PFGFTTATEF

A0A044UAC6_ONCVO PMGFTTASEV

Q59UY8_CANAL PLGFTTASEF

RAD51_YEAST PMGFVTAADF

RAD51_SCHPO PMGFTTATEY

RAD51_USTMA PMGFTTATEF

RAD51_ARATH PLGFTSASQL

RAD51_SOLLC PLGFTSASQL

RAD51_LEIMA PMGFTSAVAY

RAD51_TCRUZI PMGFTSAVVY

RAD51_Tbrusei PMGFTRATVF

RAD51_DROME PLGFLSARTF

A5K498_PLAVS NSGFCNAIDY

RAD51_PLAF NSGFCNAIDY

RADA_SULAC DIRFKTALEV

RADA_SULTO DIRFKTALEV

RADA_SULSO DIRFKTALEV

RADA_AERPE NIDFKTAYDL

RADA_DESAM GITFKTAREV

RADA_NANEQ LLEFKTAEEV

RADA_THEAC IGNFETGEEI

RADA_THEVO IGNFETGEEI

RADA_PICTO VGNFETGEEI

RADA_ARCFU IGGFESGDKV

RADA_HALSA VGGFETGATV

RADA_HALVO VGGFETGSMV

RADA_METAC IGGFETGDLV

RADA_METMA IGGFETGDLV

RADA_PYRAB LGTFMRADEY

RADA_PYRHO LGTFMRADEY

RADA_PYRFU LGTFMRADEY

RADA_PYRKO IGTFMRADEY

RADA_METTH KIDFETAFDV

RADA_METMP DLGFKSGVEL

RADA_METVO DLGFKSGIDL

RADA_METJA NLGFKSGTEV

RADA_PYRAE LHSFISALEV

RADA_PYRIL LHSFVSALEV

### Supplementary figure 6. Sequences used in Figure 4A consensus diagram

BRCA2_HUMAN/BRC1 GGSFRTASNK

BRCA2_HUMAN/BRC2 FRGFYSAHGT

BRCA2_HUMAN/BRC3 DTFFQTASGK

BRCA2_HUMAN/BRC4 LLGFHTASGK

BRCA2_HUMAN/BRC6 PPAFRIASGK

BRCA2_HUMAN/BRC7 CGIFSTASGK

BRCA2_HUMAN/BRC8 FSGFSTASGK

RAD51_HUMAN PMGFTTATEF

RAD51_MOUSE PMGFTTATEF

RAD51_CHICK PMGFTTATEF

RAD51_YEAST PMGFVTAADF

RAD51_SCHPO PMGFTTATEY

RAD51_LEIMA PMGFTSAVAY

RAD51_TCRUZI PMGFTSAVVY

RAD51_DROME PLGFLSARTF

RAD51_PLAF NSGFCNAIDY

RADA_SULSO DIRFKTALEV

RADA_PYRFU LGTFMRADEY

RADA_METVO DLGFKSGIDL

RADA_PYRIL LHSFVSALEV
